# Supplementary material for: Genomic and single-cell analyses reveal genetic signatures of swimming pattern and diapause strategy in jellyfish
Source: Nat Commun. 2024 Jul 15;15:5936. doi: 10.1038/s41467-024-49848-z (PMC11250803; doi:10.1038/s41467-024-49848-z)
Supplement: Supplementary file 1 — Supplementary Information [file 41467_2024_49848_MOESM1_ESM.pdf]

**Supplementary Information to:**

# Genomic and single-cell analyses reveal genetic signatures of swimming pattern and diapause strategy in jellyfish

**Authors:** Zhijun Dong[1,2]†*, Fanghan Wang[1,2]†, Yali Liu[2,3,4]†, Yongxue Li[1,2]†, Haiyan Yu[5], Saijun Peng[1,2], Tingting Sun[1,2], Meng Qu[2,3,4], Ke Sun[6], Lei Wang[1,2], Yuanqing Ma[7], Kai Chen[6], Jianmin Zhao[1,2]*, Qiang Lin[2,3,4]*

†Authors contributed equally to this work.

*Correspondence to: linqiang@scsio.ac.cn (Q.L.); zjdong@yic.ac.cn (Z.J.D.); jmzhao@yic.ac.cn (J.M.Z)

**Affiliations:**

[1]CAS Key Laboratory of Coastal Environmental Processes and Ecological Remediation, Yantai Institute of Coastal Zone Research, Chinese Academy of Sciences, Yantai, Shandong 264003, China.

[2]University of Chinese Academy of Sciences, Beijing 100101, China.

[3]CAS Key Laboratory of Tropical Marine Bio-Resources and Ecology, South China Sea Institute of Oceanology, Chinese Academy of Sciences, Guangzhou 510301, China.

[4]Guangdong Provincial Key Laboratory of Applied Marine Biology, South China Sea Institute of Oceanology, Chinese Academy of Sciences, Guangzhou 510301, China.

[5]College of the Environment and Ecology, Xiamen University, Xiamen 361102, China.

[6]State Key Laboratory of Primate Biomedical Research, Institute of Primate Translational Medicine, Kunming University of Science and Technology, Kunming, Yunnan 650500, China.

[7]Shandong Key Laboratory of Marine Ecological Restoration, Shandong Marine Resource and Environment Research Institute, Yantai, Shandong 264006, China.

**Supplementary Information**

**Table of Contents**

**Supplementary Notes 1-11** **Page 3-9**
**Supplementary Figures 1-19** **Page 10-30**
**Supplementary Tables 1-20** **Page 31-50**

## Supplementary Notes

### Supplementary Note 1. Genome sequencing and assembly

The genome sequencing information of are presented in Supplementary Table 1, 2, and 4. We calculated and plotted the 17-mer depth distributions of *T. rubra* and *A. coerulea*; the results are presented in Supplementary Figure 2 and Supplementary Table 3. The statistics for the assembly steps are listed Supplementary Table 5. Benchmarking Universal Single-Copy Orthologs (BUSCOs) assessment showed that our assembly captured 92.4% and 86.4% of the complete BUSCOs of *T. rubra* and *A. coerulea*, respectively. For Hi-C sequencing, the sequence interaction matrices are shown in Supplementary Figure 3, and the statistical analysis results of the chromosome assembles of *T. rubra* (2n=30) and *A. coerulea* (2n = 44) with genomic loading rates of 95.11% and 99.71 %, respectively, are summarised in Supplementary Table 6 and 7 and schematic representation of the genomic characteristics are shown in Supplementary Figure 4. Furthermore, the pseudo-chromosome syntenic relationship between *T. rubra* and *A. coerulea* was analyzed (Supplementary Figure 5). Evaluation of the genome for completeness based on BUSCO resulted in values of 92.4% and 86.4%, respectively, indicating the high completeness and accuracy of the assembly (Supplementary Table 5).

### Supplementary Note 2. Genome annotation

The results of genome annotation are summarized in Supplementary Table 8. We identified a total of 18,746 and 32,035 genes from *T. rubra* and *A. coerulea* genomes, respectively (Supplementary Table 9). In total, 90.14% and 97.13% of the predicted genes were annotated using different databases in *T. rubra* and *A. coerulea*, respectively (Supplementary Table 10). For annotation of non-coding RNAs (ncRNAs), microRNAs (miRNAs) and snRNAs were predicted using Rfam (v14.1), whereas tRNAs were screened using tRNAscan-SE (v1.3.1) and rRNA was predicted using BLASTN (v2.6.0) (Supplementary Table 11). The annotation results were evaluated using BUSCO (metazoa_odb10), and the results showed that our gene set contained 93.3% and 88.4% of complete ortholog genes of *T. rubra* and *A. coerulea*,

respectively (Supplementary Table 12), showing that our gene annotation was highly complete.

**Supplementary Note 3. Phylogenetic analysis**

To determine the phylogenetic positions of *T. rubra* and *A. coerulea*, phylogenetic analysis was conducted using whole-genome protein datasets of 17 cnidarians and one ctenophore as the outgroup. A stringent set of orthologues was identified. Alignment of these individual orthologous groups, followed by concatenation, resulted in amino acid alignment. Model prediction revealed that the JTTF model was the best-suited substitution model for concatenated alignment. RAxML was used to generate an ML tree for alignment using the best-fit model. The phylogenetic relationships between anthozoans, hydrozoans, cubozoans, and scyphozoans were consistent with those reported in previous studies based on molecular datasets. Our phylogenomic analysis also placed *T. rubra* within Hydrozoa and appeared to be a sister to *C. hemisphaerica*. The divergence times were estimated using the Markov chain Monte Carlo (MCMC) tree in PAML with calibration. Concatenated supergenes and species trees were used as input files.

**Supplementary Note 4. Expansion and contraction of gene families**

Gene expansion and contraction results for each branch of the phylogenetic tree were estimated. *T. rubra* harbored 81 significantly expanded and 278 significantly contracted gene families (Supplementary Figure 6, Supplementary Data 10). GO and KEGG enrichment analysis of contracted gene families in *T. rubra* were conducted by the GOseq R package and KOBAS software, respectively. A full list of the significantly enriched pathways is shown in Supplementary Data 11.

**Supplementary Note 5. Gene loss**

The lost gene families found in *R* language and manually searched are presented in Supplementary Data 12. A broader comparison of four vertebrates was conducted to confirm the number of genes or families associated with statocyst/otolith formation,

cilia, and nerves in compared species. GO and KEGG enrichment analysis of lost gene families in *T. rubra* were conducted by the GOseq R package and KOBAS software, respectively. All significantly enriched pathways are listed in Supplementary Data 13.

**Supplementary Note 6. Positive selection of genes**

A total of 548 PSGs were identified in *T. rubra* (FDR < 0.05) (Supplementary Data 1). The dN/dS valve was provided in the Supplementary Data 2. Notably, among these genes, we found several genes that play important roles in the statolith morphogenesis, ciliary movement, ciligenesis and some modulators that have been reported to be important for the statolith formation process (Supplementary Table 14). Genes involved in the nervous and muscular systems were also identified. GO and KEGG enrichment analyses were also conducted by the GOseq R package and KOBAS software. GO enrichment of positive selected genes (PSGs) in *T. rubra* is shown in Supplementary Data 3. Comparison of the amino acid substitutions of PSGs shown in Fig. 3b in different species were performed using MEGA-X v10.1.8, and sequences of four vertebrate species were obtained from NCBI, the GenBank accession numbers of these genes are listed in Supplementary Table 14.

**Supplementary Note 7. Transcriptome analyses**

The RNA sequencing information of two tissues (sensory organs and bell margins as controls) of four species (*C. quinquecirrha*, *R. esculentum*, *A. coerulea*, and *T. rubra*) is listed in Supplementary Data 4. Principal-component analysis (PCA) based on four species normalization genes of combined 32 transcriptomes suggested that there were more variations between species than between tissues (Supplementary Figure 7). DEGs in the tentacle bulbs of *T. rubra* with the rhopilia of the other three jellyfish were obtained using cross-species transcriptome comparisons of sensory organs (Supplementary Figure 8, Supplementary Data 5). GO and KEGG enrichment analysis of downregulated DEGs in *T. rubra* were conducted by the GOseq R package and KOBAS software, respectively. All significantly enriched pathways are listed in

Supplementary Data 6, and the top 20 significantly enriched GO terms for the downregulated DEGs in the tentacle bulb of *T. rubra* are shown for biological processes (BPs) and cellular components (CCs) in Supplementary Figure 9.

The DEGs information of the sensory organs compared with the control samples in each species is shown in Supplementary Figure 10, and 11, Supplementary Table 15 and Supplementary Data 7. The full list of significantly enrichment gene ontologies of the upregulated and downregulated DEGs in the sensory organs in each species (P-value $<0.05$) is shown in Supplementary Data 8. KEGG enrichment of cilium-related DEGs in the four jellyfish species (P-value$<0.05$) is shown in Supplementary Data 9.

**Supplementary Note 8. RNAi experiment and RT-qPCR**

The sequences of RNAi are provided in Supplementary Table 16. At the end of the RNAi experiment, polyps were observed, and the number of individuals at each stage of strobilation was recorded. The result is shown in Supplementary Figure 13a. The expression of target genes was assessed using RT-qPCR analysis, and the si-OM and si-LRR groups exhibited significant downregulation of gene expression, validating the efficacy of the siRNA treatment (Supplementary Figure 13b, $p < 0.001$; Supplementary Data 14). The RT-qPCR primer sequence of select gene are shown in Supplementary Table 17.

**Supplementary Note 9. Variation of the transcriptional expression profile of hair, neural, and muscle cells between *T. rubra* and *A. coerulea*.**

The sc-RNA sequencing information of *T. rubra* and *A. coerulea* medusa is listed in Supplementary Table 18. We generated cell atlas of medusa *T. rubra* and *A. coerulea* contain 22245 cells and 18936 cells, respectively, and the number of RNA detected and UMI per cell are shown in Supplementary Figure 14. The 18 putative cell clusters (associated markers in parentheses) with a resolution of 0.2 were assigned to 10 broad partitions and were annotated manually by combining analysis of underlying

molecular profiles and prior knowledge (Supplementary Figure 15; Supplementary Data 15). Stem/germ cells (*PCNA* and *NANOS*) were dominant in *T. rubra*, whereas glands (trypsin, chitinase, and *MUC2*) and nematoblasts (nematogalectin and *DKK3*) were dominant in *A. coerulea*. Gene expression in the hair cells of *T. rubra* and *A. coerulea* was compared and species-specific gene sets were clearly visualised. The DEGs in hair cells from *T. rubra* and *A. coerulea* are shown in Supplementary Data 16. GO enrichment analysis of the DEGs was conducted using the GOseq R package, and the full list of enriched terms is shown in Supplementary Data 17.

The list of different expressed genes (DEGs) in neural s and striated muscle between *T. rubra* and *A. coerulea* is shown in Supplementary Data 18 and 20, and GO enrichment terms are shown in Supplementary Data 19 and 21.

**Supplementary Note 10. Single-cell transcriptional profiles of cyst (Cy) formation in *T. rubra*.**

ScRNA-seq analysis was conducted on single cells sampled across five crucial life stages: medusa (Me), four-leaf structure (Ff), cyst (Cy), polyp (Po), and planula (Pl) (Supplementary Table 19). To facilitate comparative analyses across different developmental stages, we integrated the expression data from all libraries into a unified dataset and utilized the Harmony package (fast, sensitive, and accurate integration of single-cell data with Harmony) to mitigate batch effects. In total, we obtained a high-quality dataset comprising 44954 cells expressing 15478 genes, with a median of 668 genes per cell and 1521 unique molecular identifiers (UMIs) per cell, representing five critical stages of development. To construct a cell atlas, we performed a clustering analysis on the gene expression matrix, resulting in the identification of 36 distinct cell clusters. The cellular landscape was visualized using the uniform manifold approximation and projection (UMAP) technique. The 36 clusters were confidently assigned to six broad (associated markers in parentheses) cell types based on known markers and prior annotations (Fig. 5a), including stem/germ cells (*CNIWI*, *NANOS* and *PCAN*), gastroderm (*CTSZ* and *CTSB*),

epidermal/muscle cells (*MYHCKB* and *MYH2*), nematocytes (minicollagen and nematogalectin), neural cells (*SYT16* and neuocalcin), gland cells (chitinase 2 and *CELA3B*), and hair cell (myosin-VI, *MYO7A*, *PO4F3*). DEGs of *T.rubra* across different life stages are shown in Supplementary Data 25. The KEGG and GO enrichment analysess of cysts and planula in *T. rubra* (P-value < 0.05) are shown in Supplementary Data 23 and Supplementary Data 24, respectively.

## Supplementary Note 11. Whole-mount in situ hybridisation protocol

Whole-mount in situ hybridised animals that had been starved for at least 2 days were relaxed in 2% $MgCl_2$ (in 0.22 μm filtered seawater) for 5–10 min and subsequently fixed overnight at 4 °C in 4% paraformaldehyde. Thereafter, the specimens were dehydrated using a 25/50/75% MeOH series to remove undesired pigmentation and stored in 100% MeOH at -20 °C. The samples were rehydrated with a 75/50/25% MeOH series for 10 min each, followed by a 10-minute PBT wash, and then bleached in a 3% H2O2/PBT solution for 10 min. Animals were permeabilized for 10–20 min with 1 μg/ml proteinase K (Sigma) in PBT after three 10-min PBT washes. Protease digestion was stopped by a quick wash and a 10-min wash with 4 mg/ml glycine in PBT, followed by three 10-min PBT washes to remove residual glycine. The samples were washed twice with 0.1 M triethanolamine for 10 min, and then treated with 2.5 μl/ml and 5 μl/ml acetic anhydride in 0.1 M triethanolamine (pH 7.8) for 5 min each to reduce probe nonspecific binding. Hereafter, the samples were refixed overnight with 4% paraformaldehyde at 4 °C.

The fixative was thoroughly washed with three 10-min PBT washes and two 10-min 2× SSC washes. The animals in 2× SSC were transferred to a 70 °C water bath for 20 min. Prehybridization and hybridization were all carried out in hybridization oven at 57 °C. Prehybridization step was performed in hybridization buffer (50% Formamid, 5× SSC, 0.1% Tween-20, 0.1% CHAPS, 1× Denhardt's solution and 100μg/ml Heparin in DEPC water) with 0.5 mg/ml torula yeast RNA (Sigma; #R6625-25G) for 2 hours. For hybridisation, all probes denatured at 70 °C for

5 min were used at 10 ng/ml in hybridisation buffer with 0.5 mg/ml torula yeast RNA to improve signal-to-noise and hybridised for 24 hours with gentle agitation.

Unhybridized probes were washed away with pre-warmed solutions at 57 ℃ using 100% HS, 75% HS/25% 2× SSC, 50% HS/50% 2× SSC and 25% HS/75% 2× SSC for 10 min each, as well as 2× SSC with 0.1% CHAPS for two 30-min. This was followed by two 10-min washes with MAB-T (100 mM maleic acid, 150 mM NaCl, 0.1% Tween 20, pH 7.5). The samples were incubated in MAB-T with 1% BSA Fraktion V (Coolaber; # CA1381-10G) for 1 hour at RT, after which it was blocked at 4 ℃ for 2 hours in 1 ml blocking solution (80% MAB-T with 1% BSA and 20% sheep serum (Solarbio; #SL039)). The samples were incubated overnight with 1:2000 anti-DIG AP (Roche; #11093274910) in a blocking solution at 4 ℃.

The samples then were washed nine times with MAB-T for 20 min each, rinsed with NTMT (100 mM NaCl, 100 mM Tris-HCl, 50 mM MgCl2, and 0.1% Tween-20 in DEPC water, pH 9.5) for 10 min, and developed in the presence of NBT/BCIP substrate (Roche; #11175041910) at RT in the dark. The colour reaction was stopped by three PBT washes, and background staining was cleared by incubation in 70% and 100% EtOH. The samples were sequentially cleared with 80% glycerol. The primer sequences are listed in Supplementary Table 20.

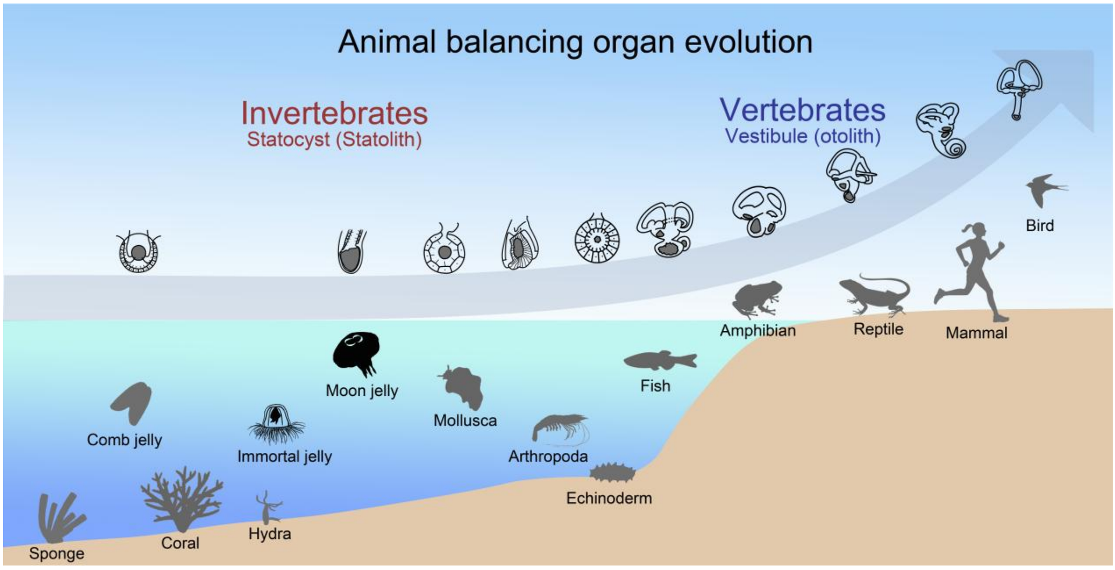


**Supplementary Figure 1.**

Balancing organs in Eumetazoa range from simple statocysts in aquatic invertebrates to complex vestibules in mammals, which comprise mass blocks of calcium crystals (statolith/otolith), proteoglycans and collagen, together with sensory hair cells mechanically influenced by the position of the mass blocks. The silhouette images of species were downloaded from BioRender. com except for echinoderm, hydra, immortal jelly and moon jelly.

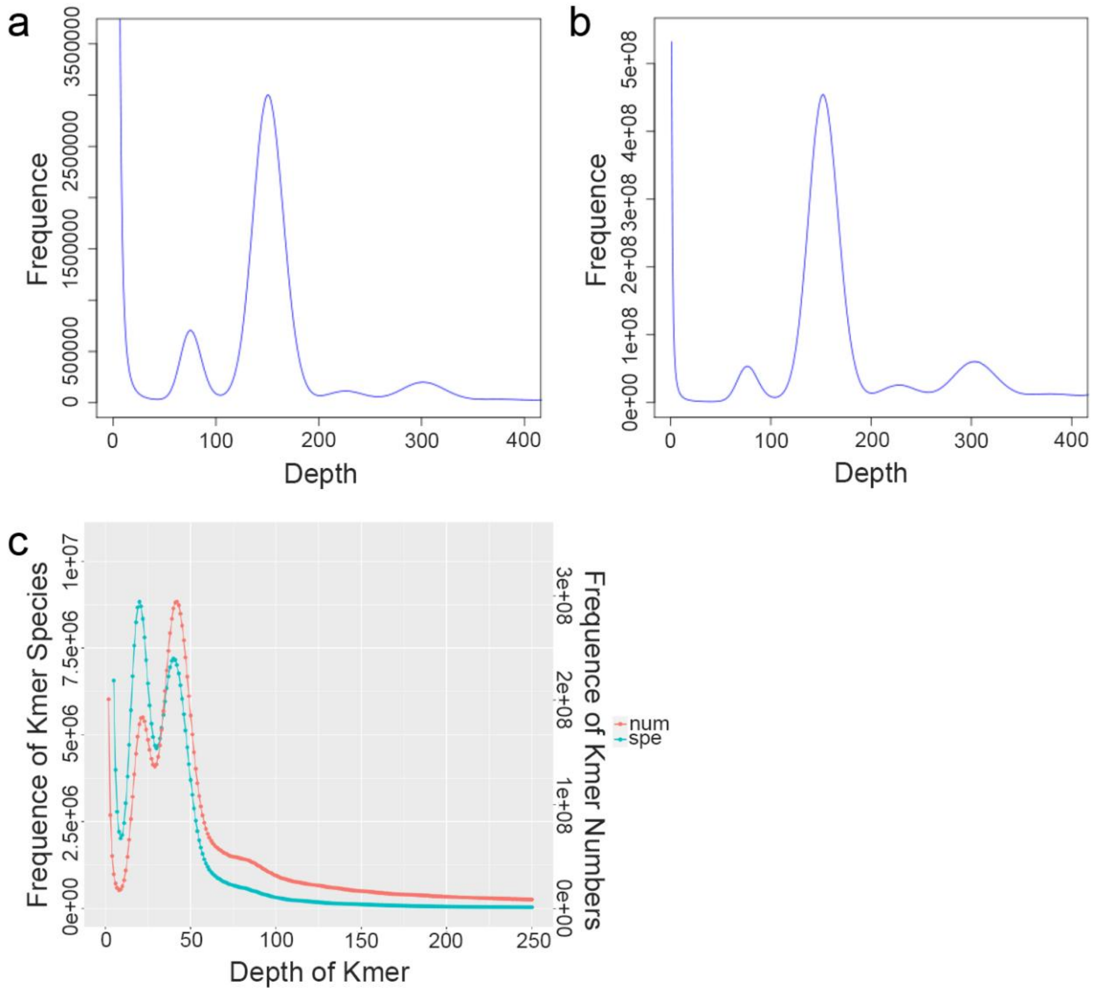


**Supplementary Figure 2.**

*K-mer* distribution of the *T.rubra* (a, b) and *A. coerulea* (c) genomes. The *K-mer* spectrum was constructed based on a 17-*mer*. The figure shows the *K-mer* spectrum of raw reads. The x-axis represents the *K-mer* depth, whereas the y-axis represents the frequence of *Kmer* species (a, c) and numbers (b, c). The K*num* of the *T.rubra* and *A. coerulea* genomes were 40,775,603,666 and 23130475928 based on the 17-mer. The K*depth* were 151 and 40, respectively.

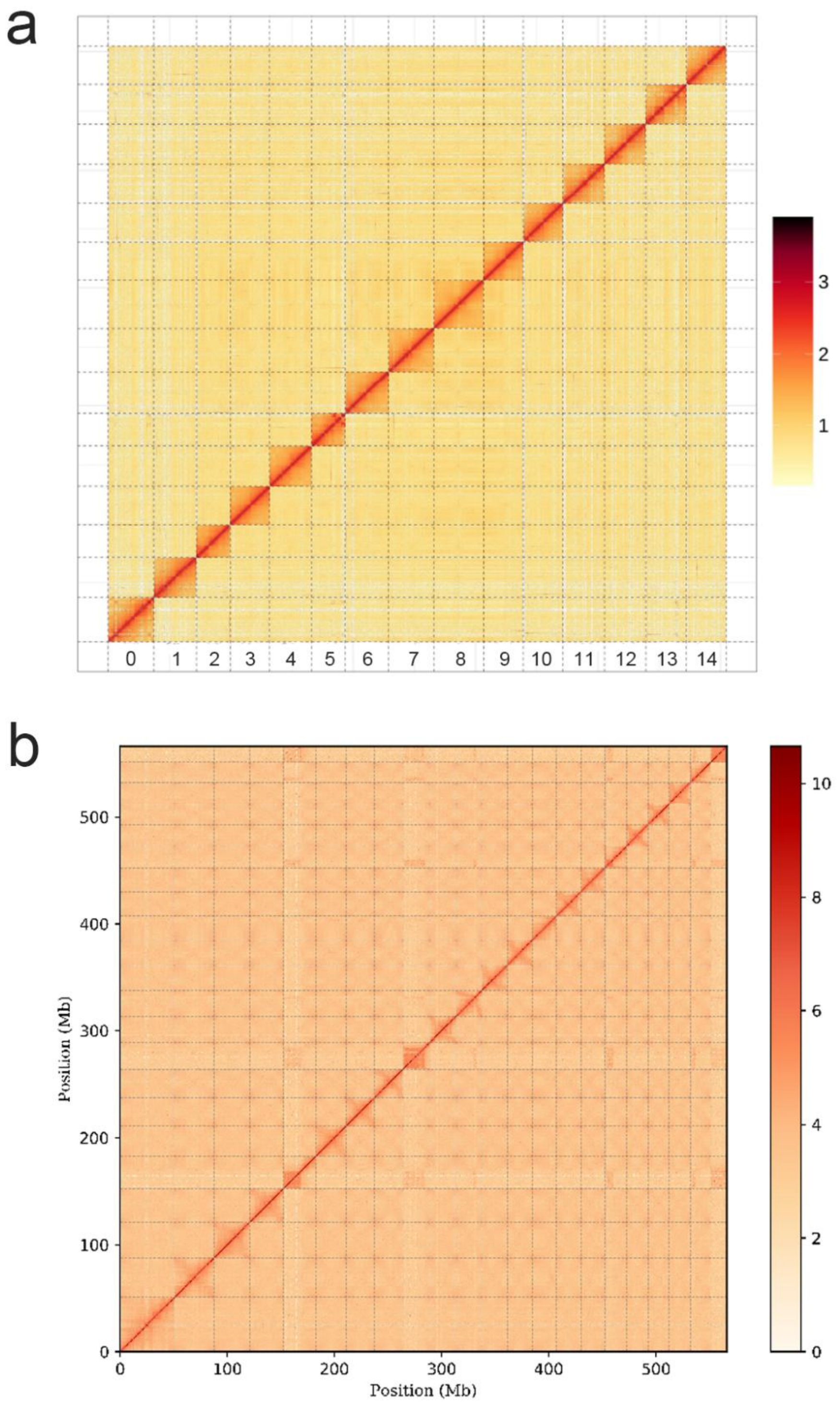


**Supplementary Figure 3.**

Hi-C contact maps of *T.rubra* (a) and *A. coerulea* (b) genome assemblies. Chromosome-level genome assemblies comprising 15 and 22 chromosome-level scaffolds, respectively.

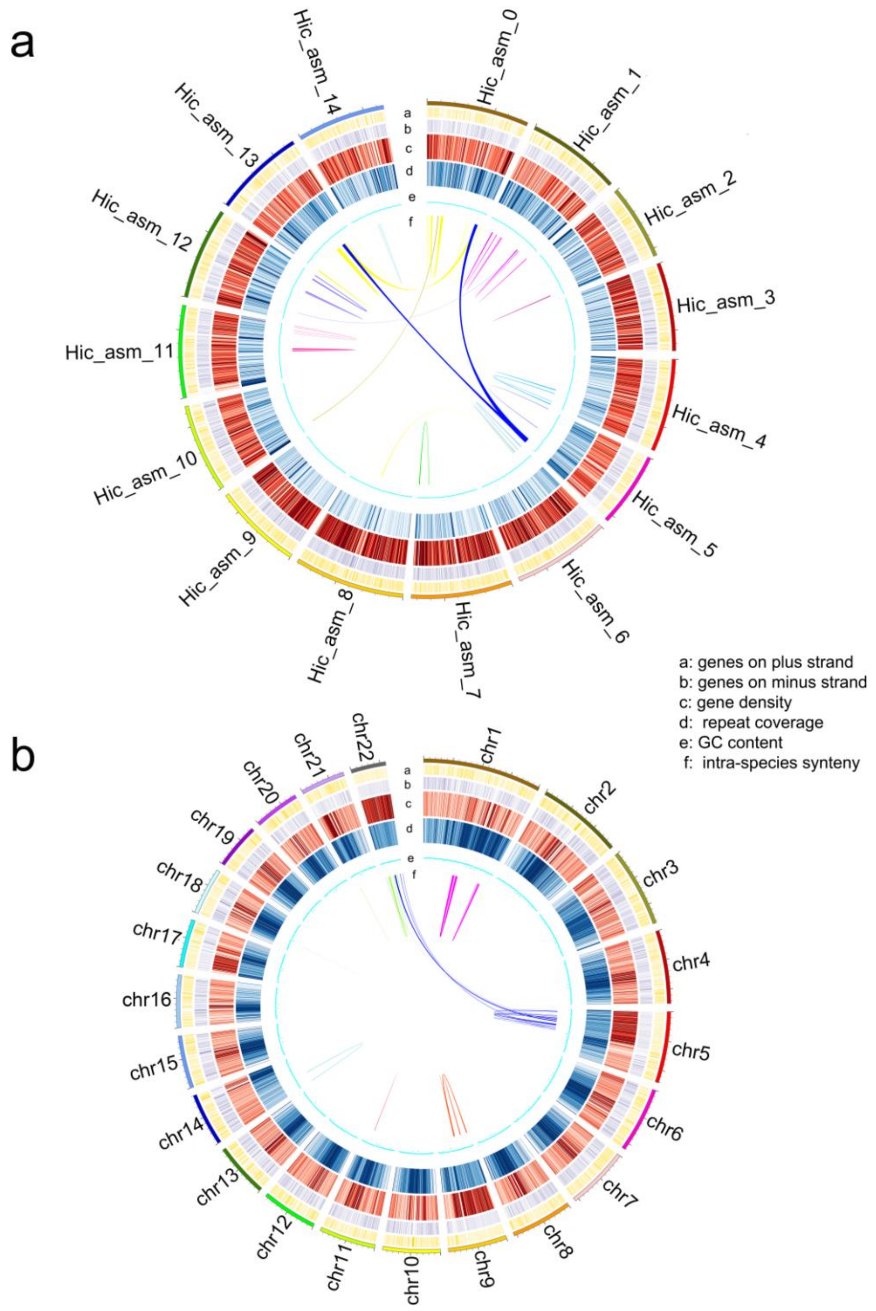


**Supplementary Figure 4.**

Schematic representation of the genomic characteristics of *T.rubra* (a) and *A. coerulea* (b). Track a: Proteincoding genes on plus strand. TrackB: Protein coding genes on minus strand. Track C: Distribution of gene density with sliding windows of 1 Mb. Higher density is shown in darker red colour. Track D: Distribution of GC content in the genome. Track E: Distribution of repeats in the genome. Track F: Schematic presentation of major interchromosomal relationships. Source data are provided as a Source Data file.

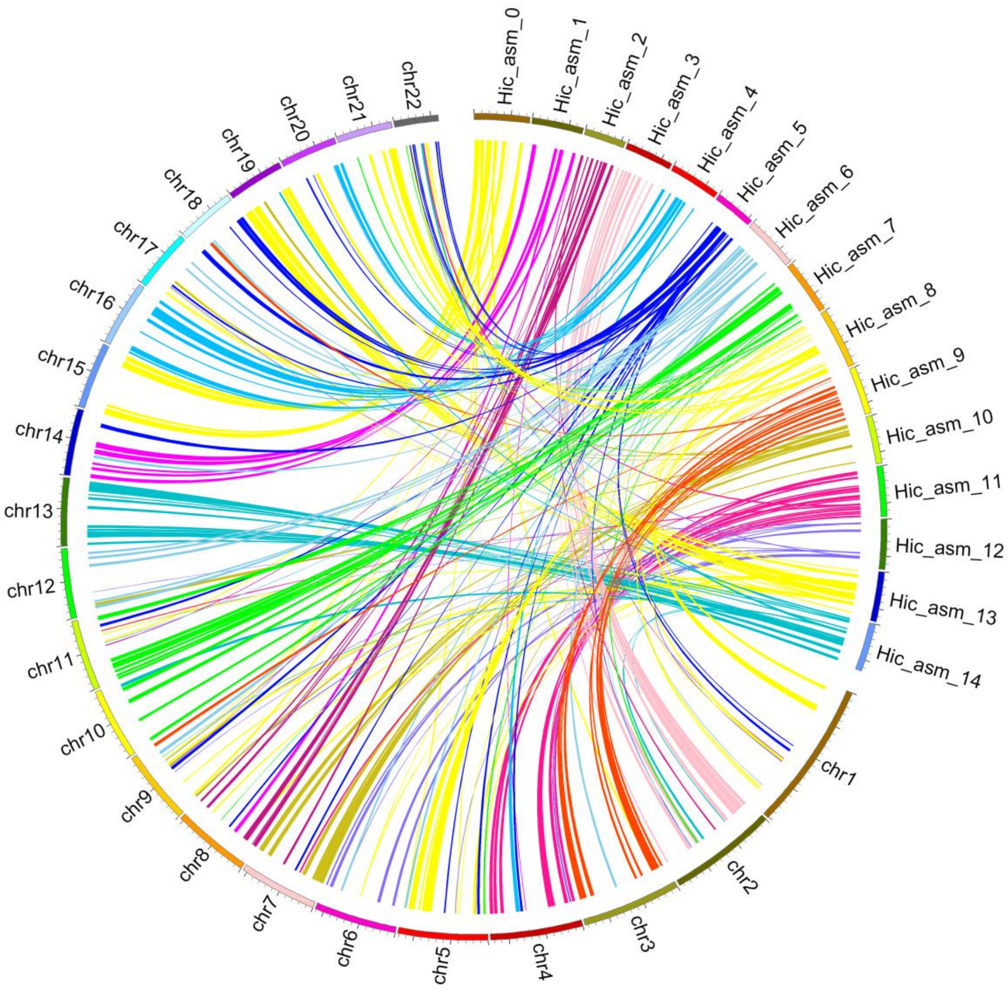


**Supplementary Figure 5.**

The chromosome synteny of *T. rubra* and *A. coerulea*. Source data are provided as a Source Data file.

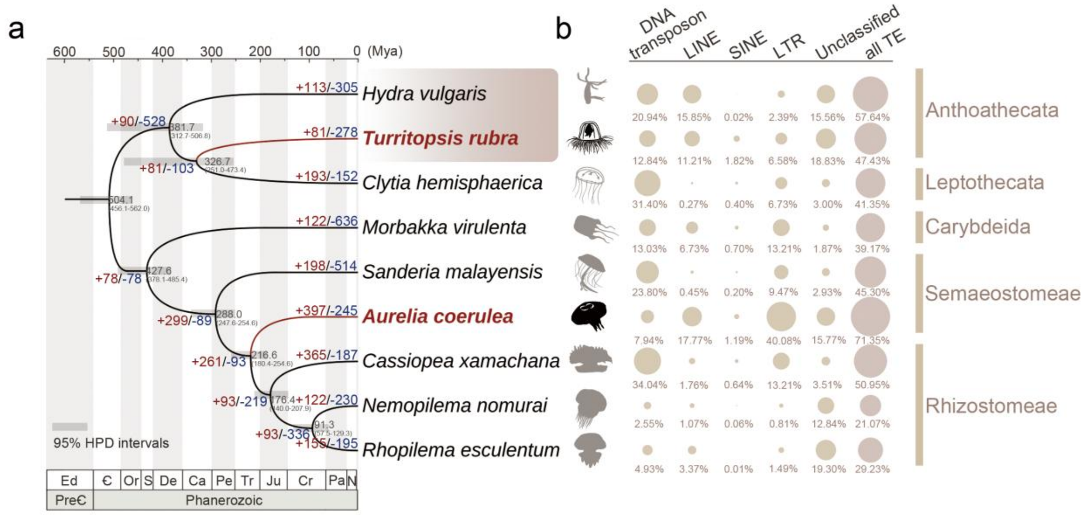


**Supplementary Figure 6.**

The numbers near each branch indicate the number of substantially expanded (red) and contracted (blue) gene families. *Hydra vulgaris* and *Turritopsis rubra,* which lack statocysts are presented in the red box. (b) Percentages of transposable elements (TEs) studied in the different jellyfish genomes studied. LINE, long interspersed element; SINE, short, interspersed element; LTR, long terminal repeats.

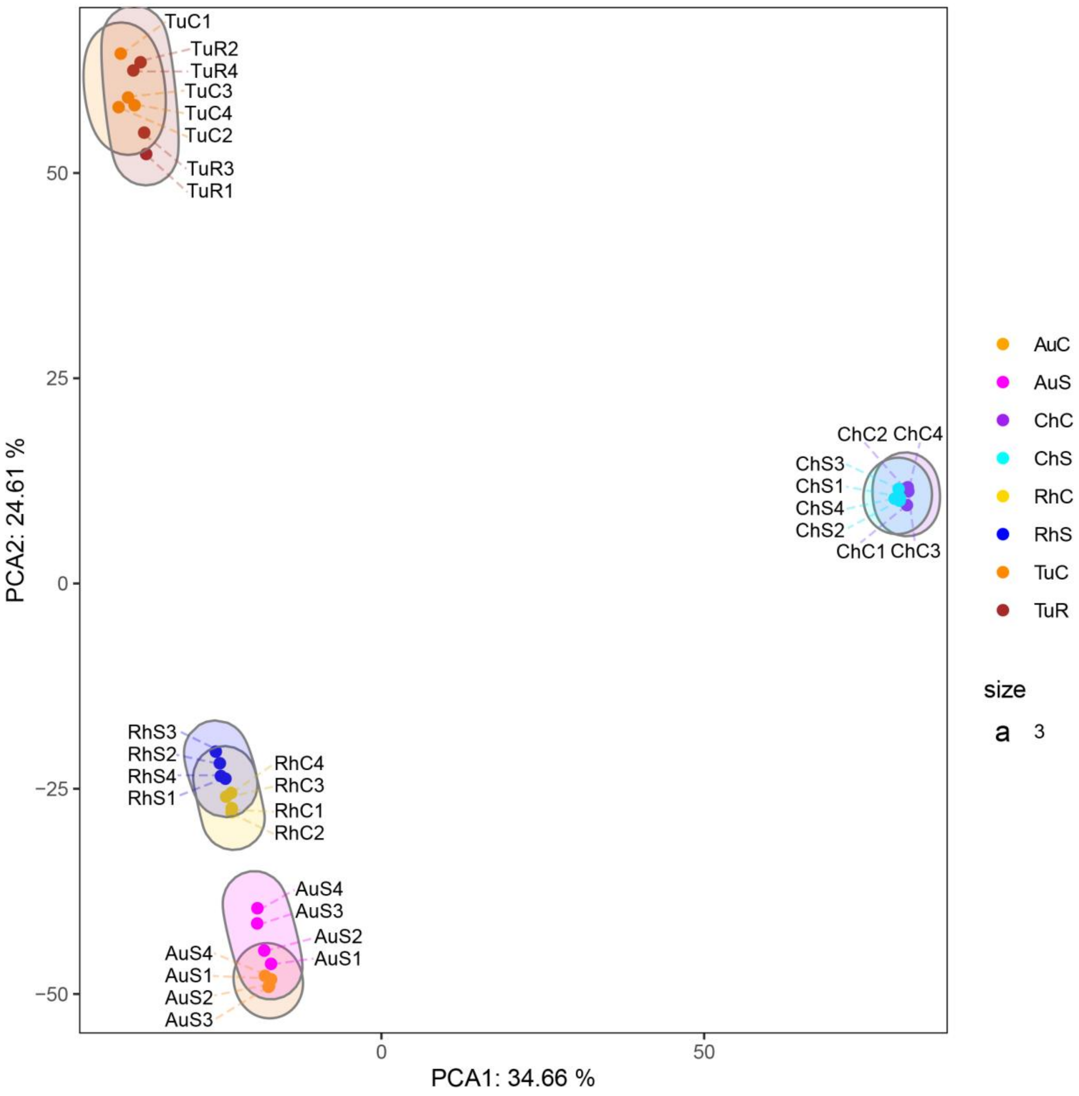


**Supplementary Figure 7.**

The principal-component analysis (PCA) clustered the 32 samples. The legend represents sample names in **Supplementary Table 15**. Source data are provided as a Source Data file.

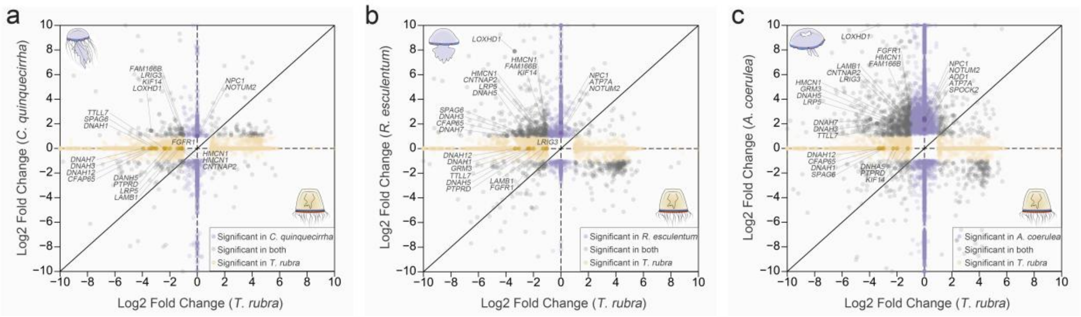


**Supplementary Figure 8.**

Volcano map of the shared and group-specific DEGs in the sensory organ and control samples of *C. quinquecirrha* (a), *R. esculentum* (b) and *A. coerulea* (c) compared to that in *T. rubra*. Yellow, purple and gray represent significantly changed DEGs in *T. rubra*, scyphozoan and both groups, respectively. Source data are provided as a Source Data file.

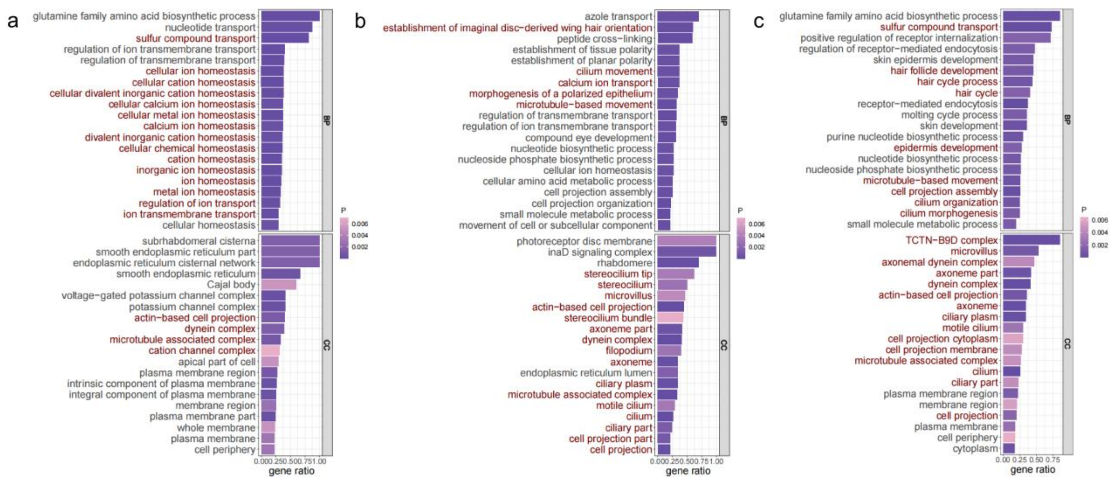


**Supplementary Figure 9.**

Top 20 enriched GO terms for downregulated DEGs in the tentacle bulb of *T. rubra* compared to three scyphozoan (a. *C. quinquecirrha*, b. *R. esculentum* and c. *A. coerulea*) are shown for biological processes (BPs) and cellular components (CCs). Categories involved in cilium are coloured in red. The enrichment was conducted using the GOseq R package and corrected $P < 0.05$ indicated significant enrichment. The complete categories are listed in Supplementary Data 6 ($P < 0.05$). Source data are provided as a Source Data file.

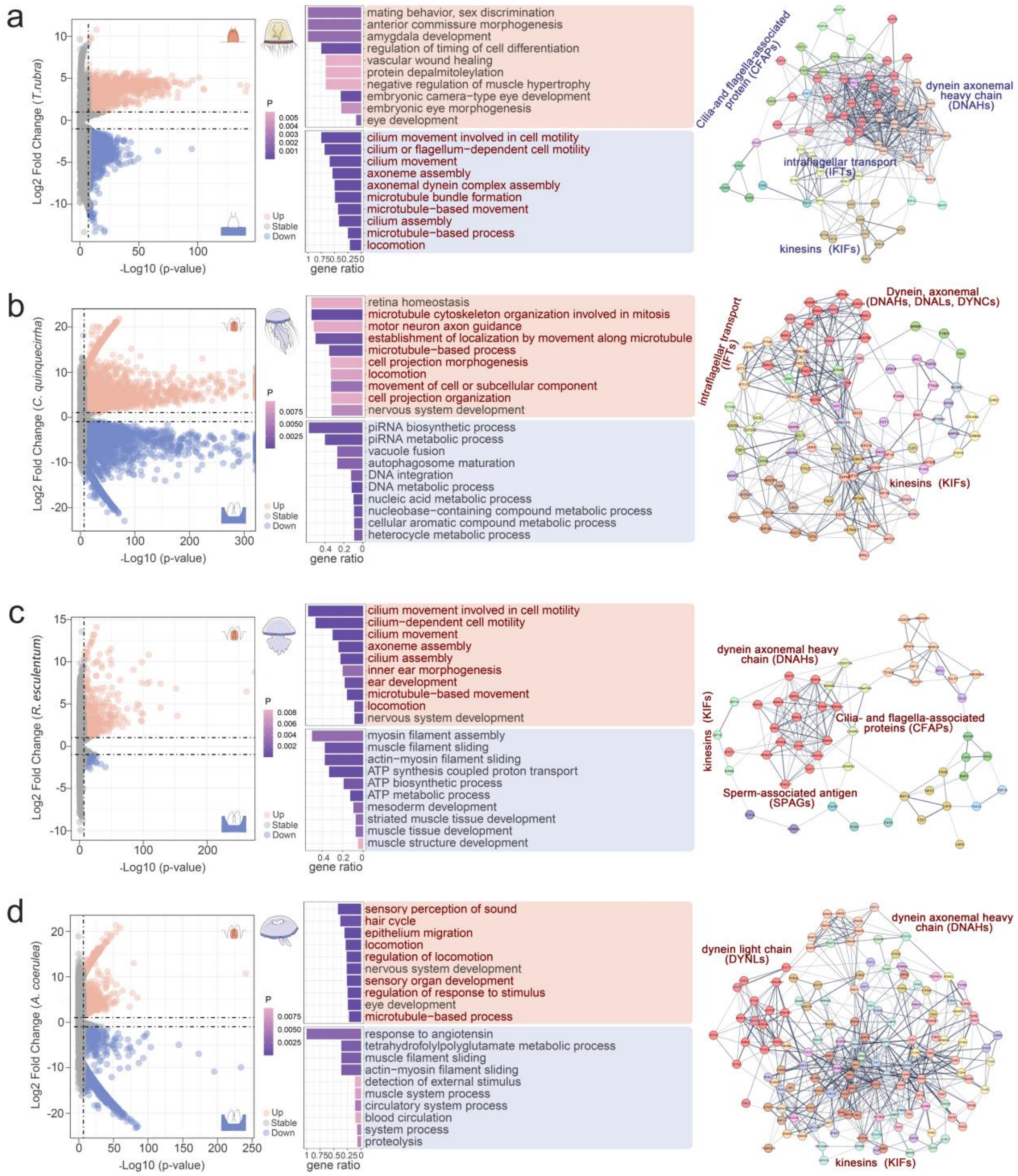


**Supplementary Figure 10.**

Volcano maps of differentially expressed genes (DEGs) in the sensory organs and control tissues of *Turritopsis rubra* (a), *Chrysaora quinquecirrha* (b), *Rhopilema esculentum* (c), and *Aurelia coerulea* (d), and significantly enriched Gene Ontology (GO) terms (biological processes, $p < 0.01$) of up-regulated and down-regulated DEGs in each species. Categories involved in cilium are coloured in red. The complete categories are listed in Supplementary Data 8 ($P < 0.05$). The interaction networks of cilium-related DEGs for each species are displayed in the right panel. The red and blue protein names in bubbles indicate up-regulated and down-regulated

proteins, respectively, in the sensory organ of each species. Edges represent protein–protein associations made using the STRING database with a medium confidence level (0.4). Coloured ellipses depict the regrouping of the closest clusters. The network of all the interactors was determined using Markov Clustering MCL (inflation parameter set to 3.0). Line thickness indicates the strength of evidence, with thicker connections representing higher confidence in the protein–protein interaction. Source data are provided as a Source Data file.

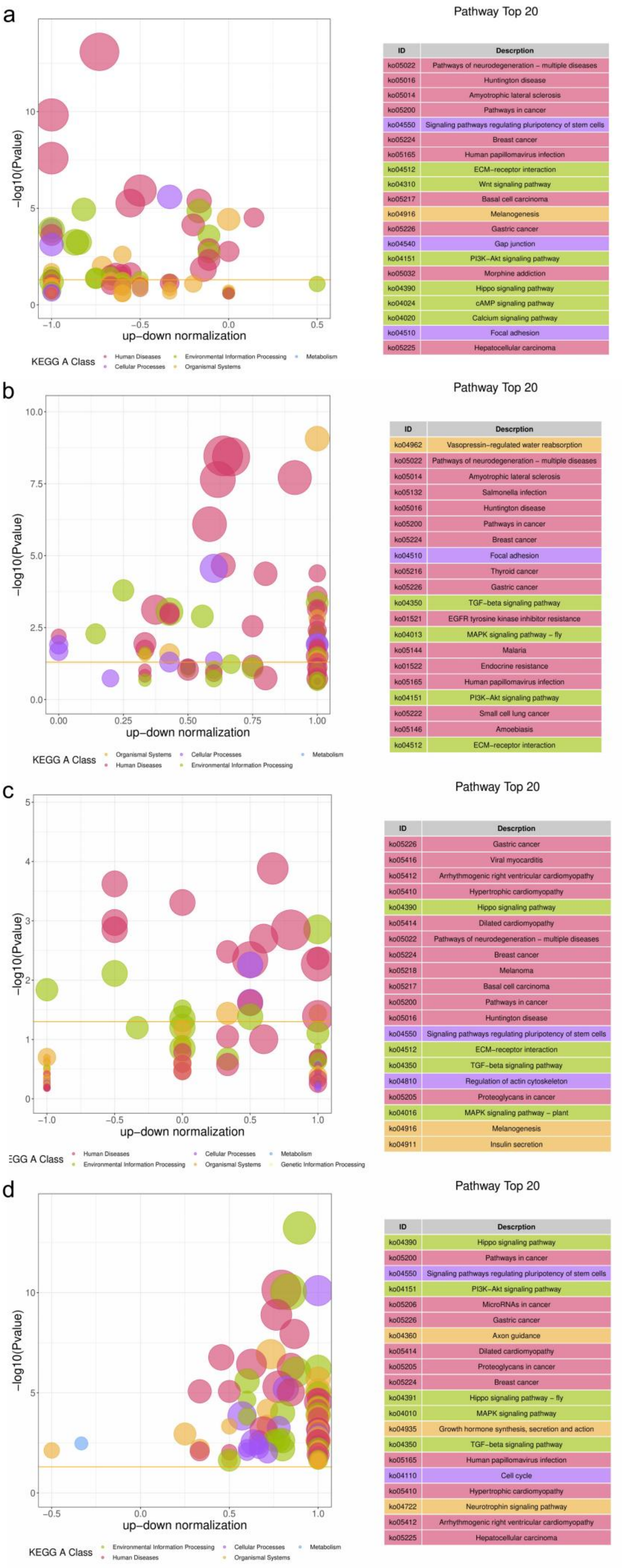


a — Pathway Top 20

| ID | Descrption |
|---|---|
| ko05022 | Pathways of neurodegeneration – multiple diseases |
| ko05016 | Huntington disease |
| ko05014 | Amyotrophic lateral sclerosis |
| ko05200 | Pathways in cancer |
| ko04550 | Signaling pathways regulating pluripotency of stem cells |
| ko05224 | Breast cancer |
| ko05165 | Human papillomavirus infection |
| ko04512 | ECM–receptor interaction |
| ko04310 | Wnt signaling pathway |
| ko05217 | Basal cell carcinoma |
| ko04916 | Melanogenesis |
| ko05226 | Gastric cancer |
| ko04540 | Gap junction |
| ko04151 | PI3K–Akt signaling pathway |
| ko05032 | Morphine addiction |
| ko04390 | Hippo signaling pathway |
| ko04024 | cAMP signaling pathway |
| ko04020 | Calcium signaling pathway |
| ko04510 | Focal adhesion |
| ko05225 | Hepatocellular carcinoma |

b — Pathway Top 20

| ID | Descrption |
|---|---|
| ko04962 | Vasopressin–regulated water reabsorption |
| ko05022 | Pathways of neurodegeneration – multiple diseases |
| ko05014 | Amyotrophic lateral sclerosis |
| ko05132 | Salmonella infection |
| ko05016 | Huntington disease |
| ko05200 | Pathways in cancer |
| ko05224 | Breast cancer |
| ko04510 | Focal adhesion |
| ko05216 | Thyroid cancer |
| ko05226 | Gastric cancer |
| ko04350 | TGF–beta signaling pathway |
| ko01521 | EGFR tyrosine kinase inhibitor resistance |
| ko04013 | MAPK signaling pathway – fly |
| ko05144 | Malaria |
| ko01522 | Endocrine resistance |
| ko05165 | Human papillomavirus infection |
| ko04151 | PI3K–Akt signaling pathway |
| ko05222 | Small cell lung cancer |
| ko05146 | Amoebiasis |
| ko04512 | ECM–receptor interaction |

c — Pathway Top 20

| ID | Descrption |
|---|---|
| ko05226 | Gastric cancer |
| ko05416 | Viral myocarditis |
| ko05412 | Arrhythmogenic right ventricular cardiomyopathy |
| ko05410 | Hypertrophic cardiomyopathy |
| ko04390 | Hippo signaling pathway |
| ko05414 | Dilated cardiomyopathy |
| ko05022 | Pathways of neurodegeneration – multiple diseases |
| ko05224 | Breast cancer |
| ko05218 | Melanoma |
| ko05217 | Basal cell carcinoma |
| ko05200 | Pathways in cancer |
| ko05016 | Huntington disease |
| ko04550 | Signaling pathways regulating pluripotency of stem cells |
| ko04512 | ECM–receptor interaction |
| ko04350 | TGF–beta signaling pathway |
| ko04810 | Regulation of actin cytoskeleton |
| ko05205 | Proteoglycans in cancer |
| ko04016 | MAPK signaling pathway – plant |
| ko04916 | Melanogenesis |
| ko04911 | Insulin secretion |

d — Pathway Top 20

| ID | Descrption |
|---|---|
| ko04390 | Hippo signaling pathway |
| ko05200 | Pathways in cancer |
| ko04550 | Signaling pathways regulating pluripotency of stem cells |
| ko04151 | PI3K–Akt signaling pathway |
| ko05206 | MicroRNAs in cancer |
| ko05226 | Gastric cancer |
| ko04360 | Axon guidance |
| ko05414 | Dilated cardiomyopathy |
| ko05205 | Proteoglycans in cancer |
| ko05224 | Breast cancer |
| ko04391 | Hippo signaling pathway – fly |
| ko04010 | MAPK signaling pathway |
| ko04935 | Growth hormone synthesis, secretion and action |
| ko04350 | TGF–beta signaling pathway |
| ko05165 | Human papillomavirus infection |
| ko04110 | Cell cycle |
| ko05410 | Hypertrophic cardiomyopathy |
| ko04722 | Neurotrophin signaling pathway |
| ko05412 | Arrhythmogenic right ventricular cardiomyopathy |
| ko05225 | Hepatocellular carcinoma |

**Supplementary Figure 11.**

Top 20 KEGG pathways for DEGs of the sensory organs and control tissues in *T. rubra* (a), *C. quinquecirrha* (b), *R. esculentum* (c) and *A. coerulea* (d). The complete categories are listed in Supplementary Data 9 ($P < 0.05$).

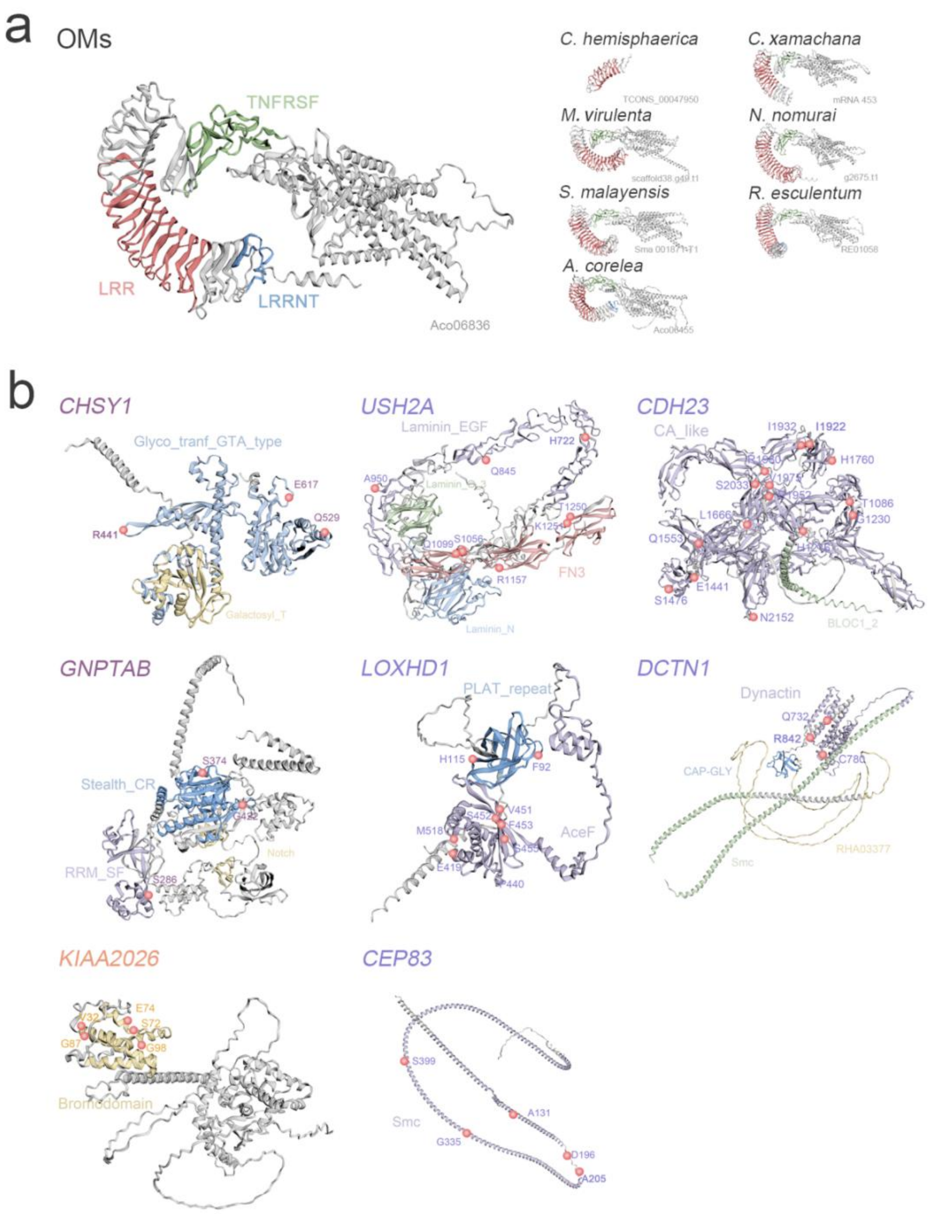


**Supplementary Figure 12.**

Predicted protein structures encoded by the lost otolith morphogenesis (OM) gene family (a) and statocyst-related positively selected genes (PSGs) (b). (a) Predicted protein structures encoded by OMs of each species revealed similar structures, with an LRR (red) and TNFRSF (green) domains (except for *Clytia hemisphaerica*), in addition to an LRRNT domain (blue) in front of the LRR in *Aurelia coerulea* and *Rhopilema esculentum*. The LRR domain plays vital role in biomineralisation. (b) Predicted protein structures encoded by some representing PSGs of Figure 2; selected amino acids are labelled on conserved domains, which are represented in different colours.

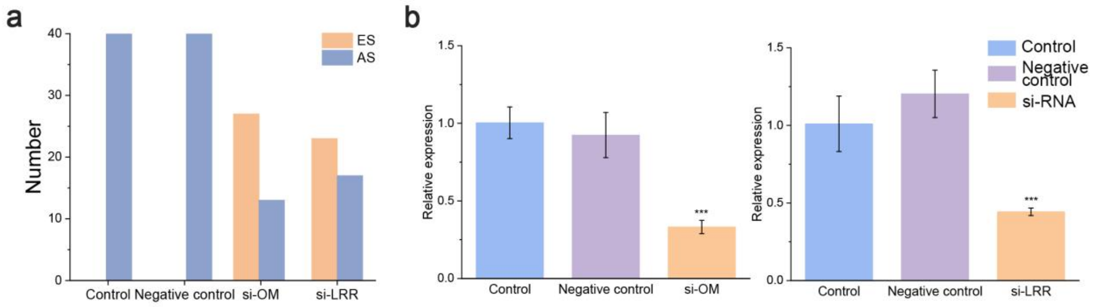


**Supplementary Figure 13**.

(a) The number of polyps under two stages of four groups. ES, early stage of strobilation; AS, advanced stage of strobilation. (b) The relative expression of target genes in the si-OM and si-LRR groups. Source data are provided as a Source Data file.

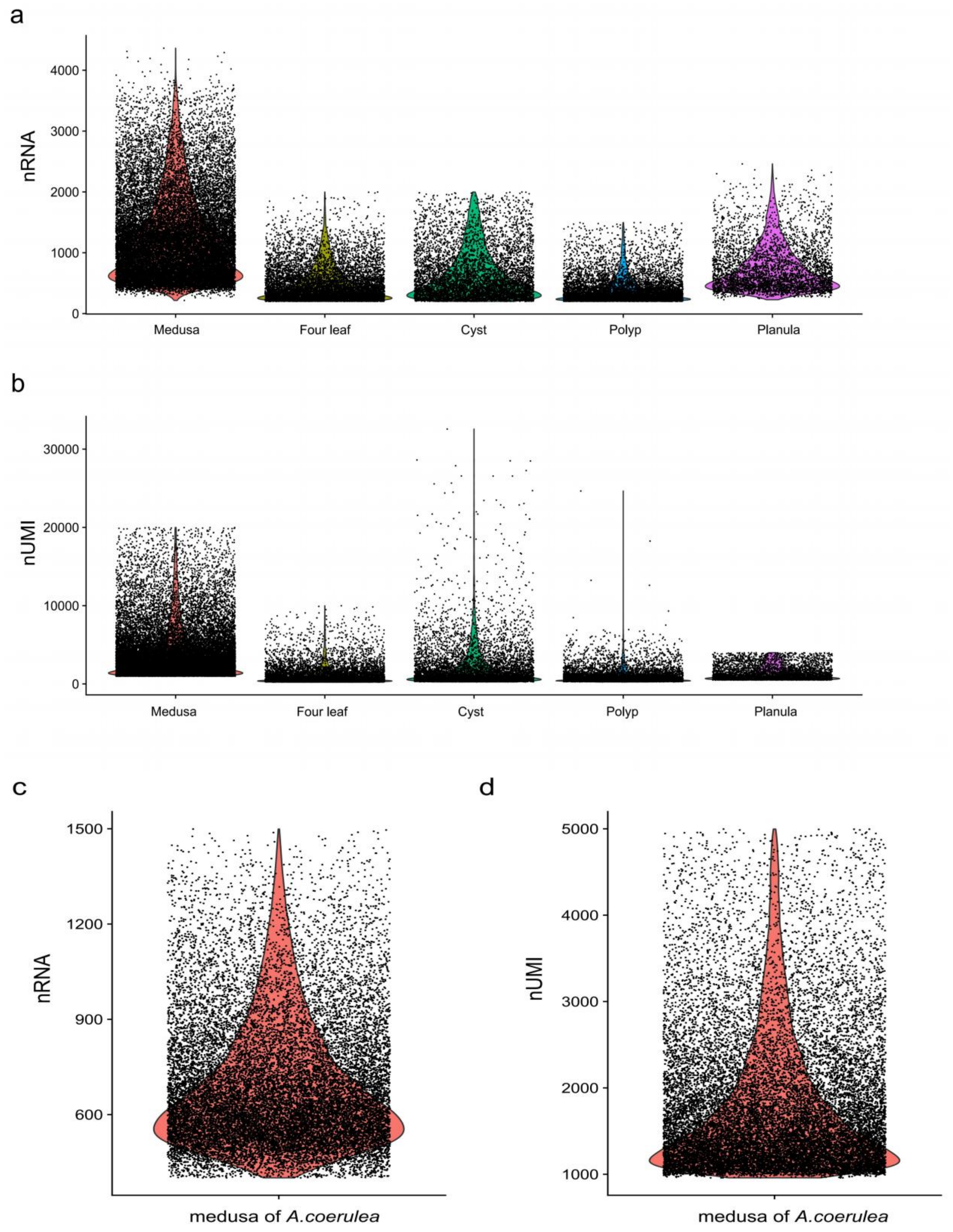


**Supplementary Figure 14.**

Violin plot showing the number of RNA detected (top) and UMI (bottom) per cell of different life stage in *T.rubra* (a, b) and medusa of *A.coerulea* (c, d).

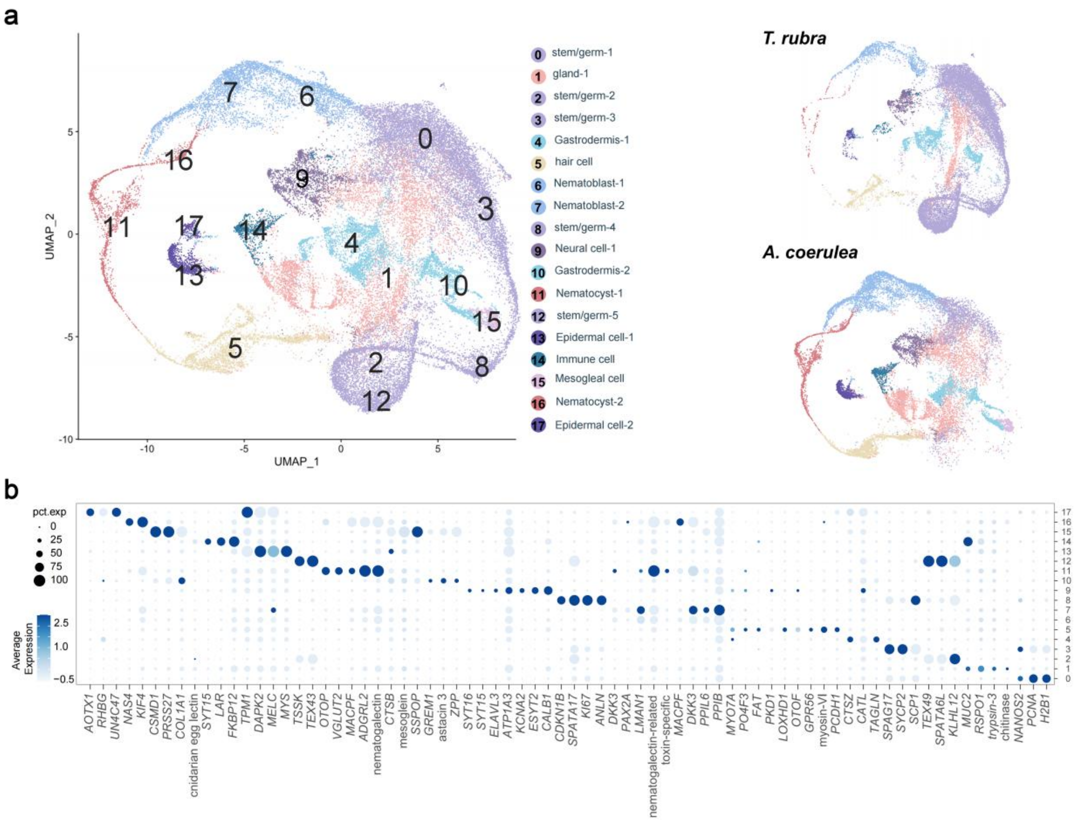


**Supplementary Figure 15.**

Cell atlas of the *T. rubra* and *A. coerulea*. (a) UMAP visualization of the merged dataset (left) and each life stage (right). coloured by cluster identity from Louvain clustering and annotated based on marker genes, cell-type colours are the same in each life stage, associated with **Figure 4**. (b) Dot plot showing expression of selected marker genes per cell type. Dot sizes represent percentages of cells within a cell type in which a given marker is detected; dot intensities represent average expression levels.

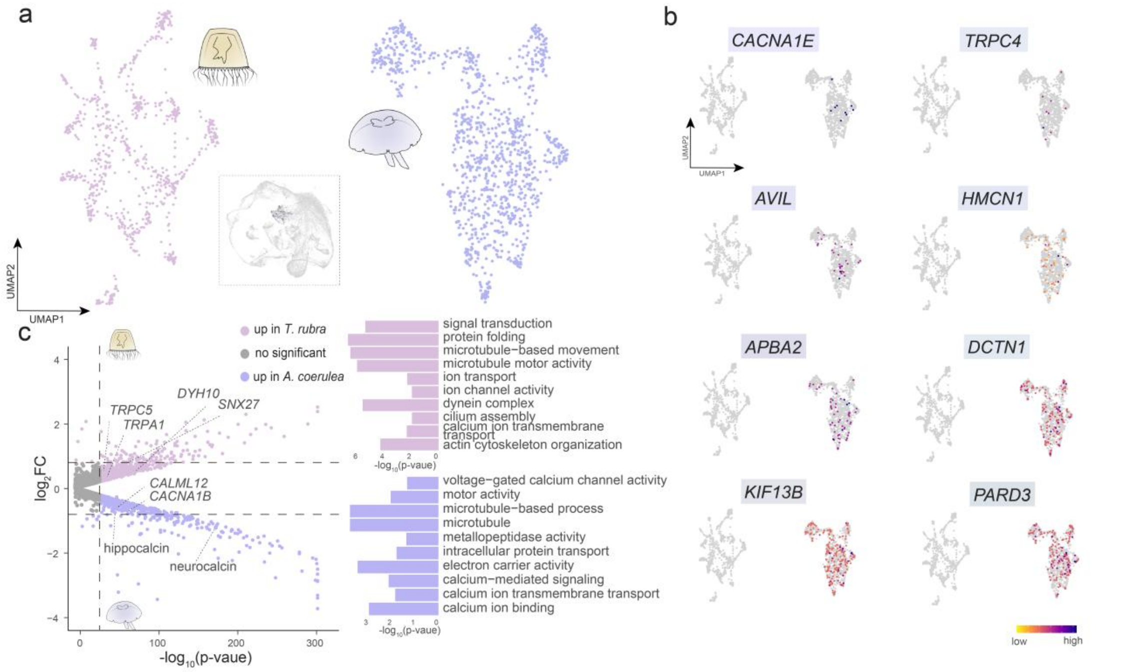


**Supplementary Figure 16.**

Cross-species comparison of neural cells in *Turritopsis rubra* and *Aurelia coerulea.* (a) Uniform Manifold Approximation and Projection (UMAP) visualisation of neural cells in the *T. rubra* and *A. coerulea* integrated medusa cell atlas. Inset: locations of the neural cell cluster of the merged UMAP plot in Figure 4a. (b) Feature plots visualising the expression of genes associated with neural development in neural cells. (c) Volcano map (left) displaying differential gene expression between the neural cells of *T. rubra* and *A. coerulea*; bar plot (right) depicts Gene Ontology (GO) analyses for differentially expressed genes (DEGs). Source data are provided as a Source Data file.

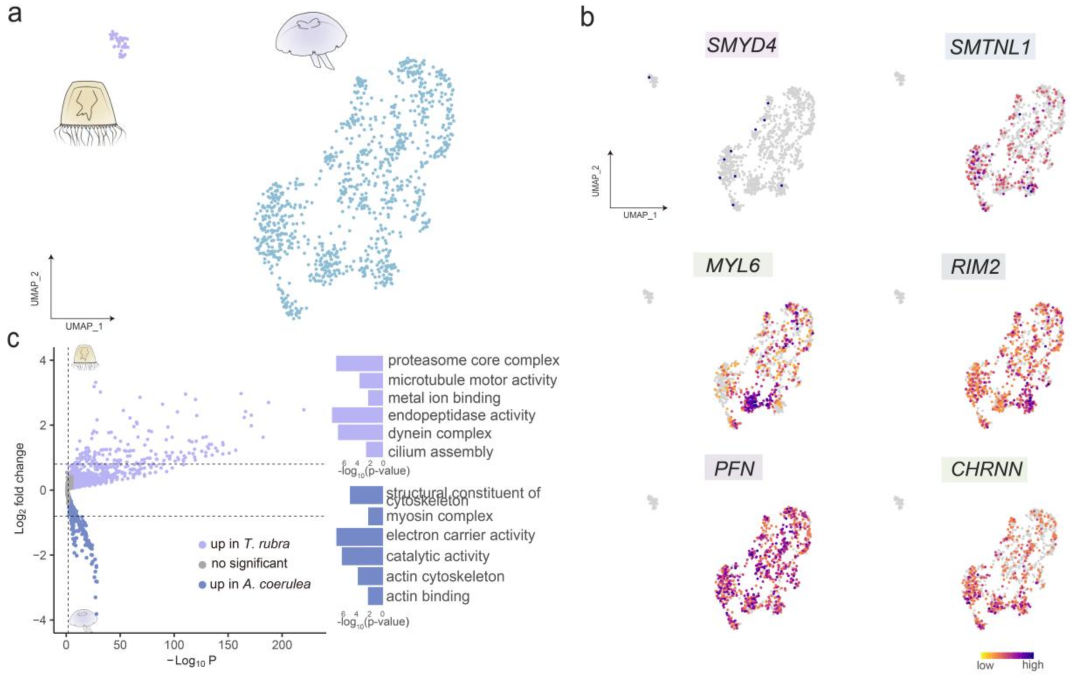


**Supplementary Figure 17.**

Cross-species comparison of striated muscle cells between *Turritopsis rubra* and *Aurelia coerulea* (a) Uniform Manifold Approximation and Projection (UMAP) visualisation of striated muscle cells in the *T. rubra* and *A. coerulea* integrated medusa cell atlas. (b) Feature plots showing the expression of genes associated with muscle contraction in striated muscle cells. (c) Volcano plot (left) depicting the differential gene expression between striated muscle cells of *T. rubra* and *A. coerulea*; bar plot (right) represents the Gene Ontology (GO) analyses for differentially expressed genes (DEGs). Source data are provided as a Source Data file.

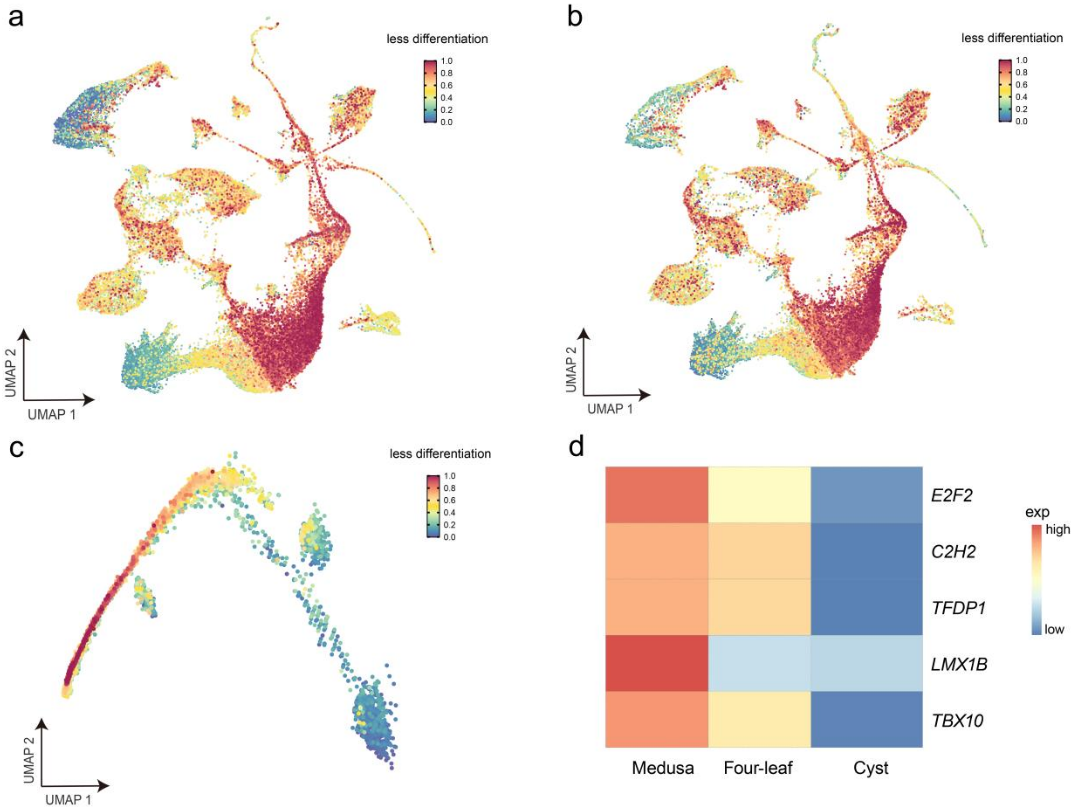


**Supplementary Figure 18.**

Analyses of the cell differentiation trajectory at different stages in *T. rubra*. (a-b) UMAP visualisation of the cell differentiation potential during the normal development stage (a) and the reverse-development stage (b) of the *T. rubra*. (c) Dynamics of differentiation potential between stem cells and nematocytes during the forward development process in *T. rubra*. (d) Heatmap showing the expression dynamics of selected transcription factors related to nematocyte differentiation during reverse development in *T. rubra*, related to Figure 5c. Source data are provided as a Source Data file.

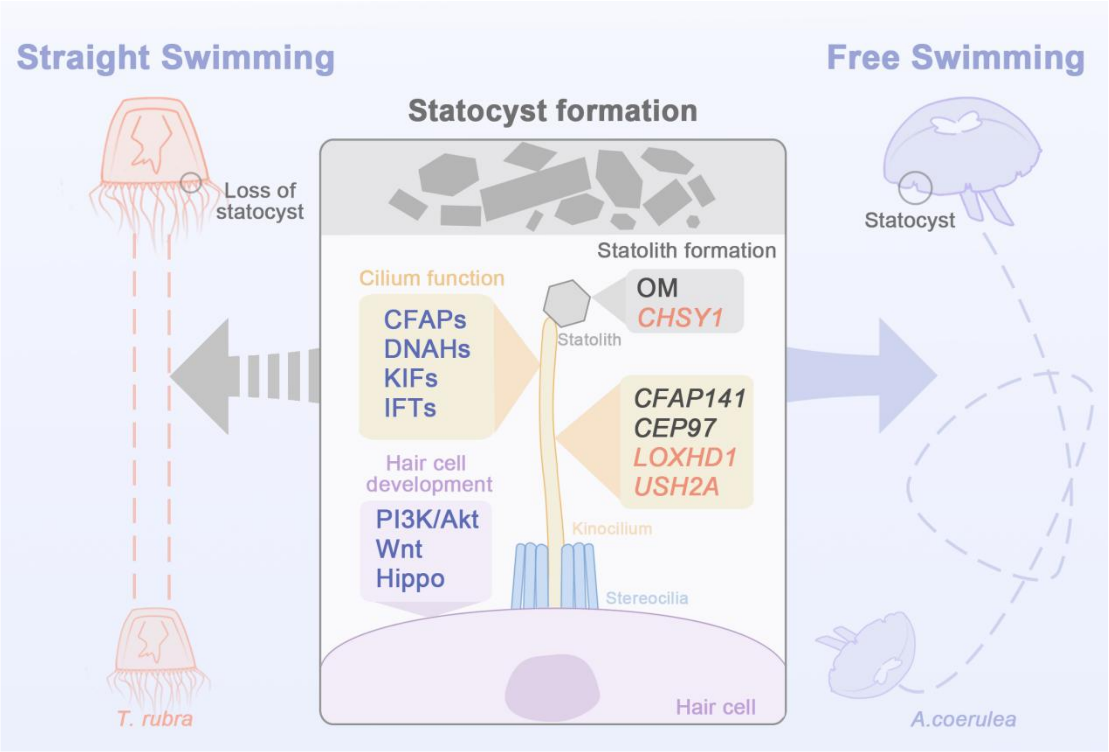


**Supplementary Figure 19.**

Genetic basis for the swimming patterns of *T. rubra* and *A. coerulea*. Lost genes (grey), positively selected genes (orange), and down-regulated or non- expressed genes and pathways (blue) in the hair cells of *T. rubra*, which are related to statocyst formation and cilium function and may result in the loss of statocyst and straight-swimming patterns in *T. rubra*.

**Supplementary Table 1. Jellyfish samples used for genome and Hi-C sequencing.**

| Species Sampling | Origin | Sampling date | Genome sequencing | Hi-C sequencing | Note |
|---|---|---|---|---|---|
| *T. rubra* | Yantai and Dongying, Shandong, China | 2021.6.5 | individual | individual | newly sequenced |
| *A. coerulea* | Yantai, Shandong, China | 2021.12.4 | individual | individual | newly sequenced |

**Supplementary Table 2. Genome sequencing data by Illumina.**

| Sample ID | Library | Data (Gb) | Depth |
|---|---|---|---|
| *T. rubra* | 350bp | 53.43 | 200× |
| *A. coerulea* | 350bp | 60.90 | 120× |

**Supplementary Table 3**. **Genome size estimation with *K-mer* distribution analysis based on a 17-*mer*.**

| Sample ID | Genome size (Mb) | Heterozygosity (%) | Repeat ratio (%) |
|---|---|---|---|
| *T. rubra* | 266.51 | 0.45 | 48.17 |
| *A. coerulea* | 521.62 | 1.43 | 56.18 |

**Supplementary Table 4. Sequencing data evaluation for Hi-C assemblies.**

| Species | Raw paired reads | Raw Base(bp) | Clean Base(bp) | Q20(%) | Q30(%) | GC Content(%) |
|---|---|---|---|---|---|---|
| *T. rubra* | 12,317,885 | 3,695,365,500 | 3,685,032,300 | 97.23 | 91.97 | 35.80 |
| *A. coerulea* | 461,577,177 | 68,707,838,179 | 68,315,728,511 | 98.80 | 95.70 | 37.30 |

**Supplementary Table 5. Summary of genome sizes and assembly statistics.**

| Species Genome size | Assembly size (bp) | Scaffold num | Scaffold N50(bp) | Contig num | Contig N50 (bp) | Complete BUSCOs (%) | Fragmented BUSCOs (%) | Missing BUSCOs (%) | Loading rate (%) |
|---|---|---|---|---|---|---|---|---|---|
| *T. rubra* | 266,862,699 | 329 | 16,943,197 | 762 | 1,187,606 | 92.40 | 1.30 | 6.30 | 95.11 |
| *A. coerulea* | 566,061,810 | 321 | 25,260,120 | 42 | 22,395,985 | 86.40 | 6.80 | 7.20 | 99.71 |

**Supplementary Table 6. Statistics of the anchored chromosomes for *T. rubra* genome.**

| | Sequeues ID | Cluster Number | Sequeues Length |
|---|---|---|---|
| | Hic_asm_0 | 57 | 18,799,587 |
| | Hic_asm_1 | 42 | 17,348,683 |
| | Hic_asm_2 | 19 | 13,911,814 |
| | Hic_asm_3 | 15 | 16,302,533 |
| | Hic_asm_4 | 20 | 17,136,266 |
| | Hic_asm_5 | 27 | 13,858,914 |
| | Hic_asm_6 | 24 | 17,883,988 |
| *T. rubra* | Hic_asm_7 | 20 | 18,556,697 |
| | Hic_asm_8 | 13 | 20,379,689 |
| | Hic_asm_9 | 16 | 16,246,515 |
| | Hic_asm_10 | 30 | 16,356,653 |
| | Hic_asm_11 | 35 | 17,031,648 |
| | Hic_asm_12 | 36 | 16,943,197 |
| | Hic_asm_13 | 46 | 16,806,440 |
| | Hic_asm_14 | 48 | 16,243,415 |

**Supplementary Table 7. Statistics of the anchored chromosomes for *A.coerulea* genome**.

| | Superscaffold | Number of Contigs | Length of Contigs | Length of Superscaffold |
|---|---|---|---|---|
| *A.coerulea* | chr1 | 4 | 50,743,788 | 50,745,288 |
| | chr2 | 1 | 36,548,555 | 36,548,555 |
| | chr3 | 4 | 33,598,094 | 33,599,594 |
| | chr4 | 4 | 31,316,877 | 31,318,377 |
| | chr5 | 1 | 30,607,789 | 30,607,789 |
| | chr6 | 1 | 28,066,466 | 28,066,466 |
| | chr7 | 1 | 26,734,379 | 26,734,379 |
| | chr8 | 1 | 26,296,151 | 26,296,151 |
| | chr9 | 2 | 25,259,620 | 25,260,120 |
| | chr10 | 1 | 24,443,289 | 24,443,289 |
| | chr11 | 2 | 24,105,683 | 24,106,183 |
| | chr12 | 1 | 23,738,836 | 23,738,836 |
| | chr13 | 2 | 23,151,249 | 23,151,749 |
| | chr14 | 4 | 22,688,858 | 22,690,358 |
| | chr15 | 2 | 22,541,530 | 22,542,030 |
| | chr16 | 1 | 22,395,985 | 22,395,985 |
| | chr17 | 1 | 20,565,505 | 20,565,505 |
| | chr18 | 1 | 20,011,490 | 20,011,490 |
| | chr19 | 1 | 19,548,997 | 19,548,997 |
| | chr20 | 3 | 19,537,659 | 19,538,659 |
| | chr21 | 2 | 19,094,960 | 19,095,460 |
| | chr22 | 2 | 15,066,050 | 15,066,550 |

**Supplementary Table 8. Statistics of TEs in *T. rubra* and *A.coerulea* genomes.**

| Species | Type | Denovo+Repbase Length(bp) | Rate (%) | TE proteins Length(bp) | Rate (%) | Combined TEs Length(bp) | Rate (%) |
|---|---|---|---|---|---|---|---|
| | DNA | 30,634,570 | 11.48 | 11,144,987 | 4.18 | 34,270,163 | 12.84 |
| | LINE | 25,796,510 | 9.67 | 11,973,960 | 4.49 | 29,915,257 | 11.21 |
| | SINE | 4,869,953 | 1.82 | 0 | 0.00 | 4,869,953 | 1.82 |
| *T. rubra* | LTR | 16,260,095 | 6.09 | 2,939,498 | 1.10 | 17,550,578 | 6.58 |
| | Other | 0 | 0.00 | 0 | 0.00 | 0 | 0.00 |
| | Unknown | 50,261,822 | 18.83 | 0 | 0.00 | 50,261,822 | 18.83 |
| | Total | 120,603,297 | 45.19 | 25,944,876 | 9.72 | 126,581,914 | 47.43 |
| | DNA | 45,886,648 | 7.97 | 707,009 | 0.12 | 45,727,839 | 7.94 |
| | LINE | 96,396,367 | 16.75 | 20,206,690 | 3.51 | 102,296,994 | 17.77 |
| | SINE | 6,859,988 | 1.20 | 0 | 0.00 | 6,853,467 | 1.19 |
| *A.coerulea* | LTR | 232,566,099 | 40.41 | 9,031,961 | 1.57 | 230,701,339 | 40.08 |
| | Other | 0 | 0.00 | 0 | 0.00 | 0 | 0.00 |
| | Unknown | 90,804,249 | 15.77 | 0 | 0.00 | 90,778,823 | 15.77 |
| | Total TE | 408,176,930 | 70.92 | 29,940,888 | 5.20 | 410,702,814 | 71.35 |

**Supplementary Table 9. Statistics of the gene prediction in *T. rubra* and *A.coerulea*.**

| Species | Gene set | Software | Species | Number |
|---|---|---|---|---|
| *T. rubra* | *De novo* | Augustus | - | 16,812 |
| | | GlimmerHMM | - | 27,384 |
| | | SNAP | - | 22,730 |
| | | Geneid | - | 5,765 |
| | | Genscan | - | 12,883 |
| | Homolog | Blast and Genewise | *Aurelia aurita* | 20,506 |
| | | | *Clytia hemisphaerica* | 25,304 |
| | | | *Hydra vulgaris* | 17,557 |
| | | | *Morbakka virulenta* | 15,451 |
| | | | *Pocillopora damicornis* | 18,625 |
| | | | *Rhopilema esculentum* | 21,438 |
| | RNAseq | PASA | - | 48,492 |
| | | Cufflinks | - | 46,749 |
| | Intergration | EVM | - | 18,746 |
| *A.coerulea* | *De novo* | Augustus | - | 51,133 |
| | | Genscan | - | 49,597 |
| | | GlimmHMM | - | 79,009 |
| | Homolog | Exonerate | *Aurelia aurita* | 76,379 |
| | | | *Aurelia* sp1 | 86,080 |
| | | | *Cassiopea xamachana* | 36,171 |
| | | | *Chrysaora quinquecirrha* | 37,770 |
| | | | *Hydra vulgaris* | 17,611 |
| | | | *Rhopilema esculentum* | 32,353 |
| | RNAseq | PASA | - | 6,658 |
| | Intergration | MAKER | - | 32,035 |

**Supplementary Table 10. Statistics of functional annotation of protein-coding genes in *T. rubra* and *A.coerulea*.**

| Species | Annotation database | Number | Percent(%) |
|---|---|---|---|
| *T. rubra* | Swissprot | 12,770 | 68.12 |
| | Nr | 16,890 | 90.10 |
| | KEGG | 13,385 | 71.40 |
| | InterPro | 16,674 | 88.95 |
| | GO | 7,805 | 41.64 |
| | Pfam | 12,322 | 65.73 |
| | Annotated | 16,898 | 90.14 |
| | Unannotated | 1,848 | 9.86 |
| *A.coerulea* | InterPro | 24,625 | 76.87 |
| | GO | 13,592 | 42.43 |
| | KEGG_ALL | 30,628 | 95.61 |
| | KEGG_KO | 8,724 | 27.23 |
| | Swissprot | 21,001 | 65.56 |
| | TrEMBL | 30,979 | 96.70 |
| | NR | 30,891 | 96.43 |
| | Annotated | 31,116 | 97.13 |
| | Unannotated | 919 | 2.87 |

**Supplementary Table 11. Statistics of the non-coding RNA of *T. rubra* and *A.coerulea* genomes.**

| Species | Type | | Copy | Average length(bp) | Total length(bp) | % of genome |
|---|---|---|---|---|---|---|
| *T. rubra* | miRNA | | 125 | 116.00 | 14,531 | 0.0050 |
| | tRNA | | 8,425 | 75.00 | 629,943 | 0.2406 |
| | rRNA | rRNA | 496 | 98.20 | 48,709 | 0.0180 |
| | | 18S | 55 | 189.31 | 10,412 | 0.0039 |
| | | 28S | 8 | 126.50 | 1,012 | 0.0004 |
| | | 5.8S | 0 | 0.00 | 0 | 0.0000 |
| | | 5S | 433 | 86.11 | 37,285 | 0.0140 |
| | snRNA | snRNA | 747 | 128.43 | 95,935 | 0.0359 |
| | | CD-box | 43 | 131.40 | 5,650 | 0.0021 |
| | | HACA-box | 16 | 185.12 | 2,962 | 0.0011 |
| | | splicing | 676 | 126.34 | 85,406 | 0.0320 |
| *A.coerulea* | miRNA | | 65 | 38.43 | 2,498 | 0.0004 |
| | tRNA | | 2,341 | 75.19 | 176,034 | 0.0306 |
| | rRNA | rRNA | 630 | 1,741.31 | 1,097,027 | 0.1906 |
| | | 18S | 187 | 1,793.33 | 335,353 | 0.0583 |
| | | 28S | 183 | 3,999.16 | 731,847 | 0.1271 |
| | | 5S | 260 | 114.71 | 29,827 | 0.0052 |
| | snRNA | snRNA | 39 | 149.43 | 5,828 | 0.0010 |
| | | CD-box | 2 | 218.00 | 436 | 0.0001 |
| | | HACA-box | 0 | 0.00 | 0 | 0.0000 |
| | | splicing | 37 | 145.72 | 5,392 | 0.0009 |
| | | scaRNA | 0 | 0.00 | 0 | 0.0000 |

**Supplementary Table 12. BUSCO evaluation of annotated results.**

| n=954 | Percentages(%) | |
|---|---|---|
| | *T. rubra* | *A. coerulea* |
| Complete BUSCOs | 93.3 | 88.3 |
| Complete and single-copy BUSCOs | 88.4 | 87.9 |
| Complete and duplicated BUSCOs | 4.9 | 0.5 |
| Fragmented BUSCOs | 1.6 | 4.7 |
| Missing BUSCOs | 5.1 | 6.9 |

**Supplementary Table 13. Comparison of genome assemblies of *Turritopsis* and *Aurelia* with published genomic statistics.**

| Species | ***T. rubra*** | *T. rubra* | *T.dohrnii* | *T. dohrnii* | ***A. coerulea*** | *Aurelia* sp1 | *A. aurita* （Atlantic） | *A. aurita* (Pacific) |
|---|---|---|---|---|---|---|---|---|
| Assembly size (bp) | **266.86** | 210.00 | 390.00 | 435.92 | **566.06** | 713.00 | 377.00 | 429.00 |
| Contig num | **762** | 53,262 | 68,044 | 891 | **42** | 67,005 | 170,088 | 213,756 |
| contig N50(bp) | **1,187,606** | 3,457 | 7,666 | 747,194 | **22,395,985** | 20k | 2,627 | 2,665 |
| Scaffold num | **329** | 9,508 | 74,829 | — | **22** | 16,793 | 2,710 | 7,744 |
| scaffold N50(bp) | **16,943,197** | 71,856 | 10,419 | — | **25,260,120** | 124K | 1.04M | 0.2M |
| GC Content(%) | **34.26** | 34.00 | 34.50 | 34.70 | **37.34** | 32.60 | 37.10 | 37.60 |
| TE rate(%) | **47.43** | 39.45 | 50.78 | 60.35 | **73.14** | 49.50 | 44.67 | 44.03 |
| Complete BUSCOs (%) | **92.40** | 88.78 | 78.88 | 90.40 | **86.40** | — | 72.20 | 49.00 |
| Gene num | **18,746** | 9,324 | 17,468 | 23,314 | **32,035** | 29,964 | 28,625 | 30,166 |
| Genome coverage | **200×** | 96× | 95× | 219.5× | **120×** | — | 90× | 90× |
| Assembly level | **chromosome** | scaffold | scaffold | contig | **chromosome** | scaffold | scaffold | scaffold |
| Reference | **This study** | Pascual-Torner et al., 2022 | | Hasegawa et al., 2023 | **This study** | Gold et al, 2019 | Khalturin et al.,2019 | |

**Supplementary Table 14. GenBank accession numbers of statocyst related PSGs protein sequences of four vertebrate species.**

| Gene | Species | GenBank accession number |
|---|---|---|
| *CHSY1* | *D. rerio* | NP_997843 |
| | *G. gallus* | XP_015147744 |
| | *M. musculus* | NP_001074632 |
| | *H. sapiens* | AAQ88893 |
| *USH2A* | *D. rerio* | XP_009291422.1 |
| | *G. gallus* | XP_015139379.2 |
| | *M. musculus* | AAZ23164.1 |
| | *H. sapiens* | KAI4084958.1 |
| *CDH23* | *D. rerio* | NP_999974.1 |
| | *G. gallus* | XP_421595.5 |
| | *M. musculus* | NP_075859.2 |
| | *H. sapiens* | NP_071407.4 |
| *DCTN1* | *D. rerio* | XP_021336434.1 |
| | *G. gallus* | XP_040555015.1 |
| | *M. musculus* | NP_001185795.1 |
| | *H. sapiens* | NP_004073.2 |
| *CEP83* | *D. rerio* | NP_001340860.1 |
| | *G. gallus* | XP_015139570.1 |
| | *M. musculus* | NP_084128.2 |
| | *H. sapiens* | NP_057206.2 |
| *KIAA2026* | *D. rerio* | XP_005173914.1 |
| | *G. gallus* | XP_004949130.2 |
| | *M. musculus* | NP_766424.2 |
| | *H. sapiens* | NP_001017969.2 |

**Supplementary Table 15. Number of DEGs of the sensory organs compared with control samples in each species (Q<0.01).**

| Species | Up | Down | Total |
|---|---|---|---|
| *T. rubra* | 1,303 | 3,335 | 4,638 |
| *C. quinquecirrha* | 4,796 | 5,140 | 9,936 |
| *R. esculentum* | 1,048 | 413 | 1,461 |
| *A. coerulea* | 3,698 | 4,032 | 7,730 |

**Supplementary Table 16. The sequences of RNAi used in the study.**

| SiRNA | Number | Sequence (5'-3') |
|---|---|---|
| Si-OM | 1 | CTATCGATCTCACGTTGAA |
| | 2 | GGCTACAACAGGCAAACAA |
| | 3 | GGCAACGACTTCAAAGGAA |
| Si-LRR | 1 | CCTACAACGAAACAGGATA |
| | 2 | CCAGACTTTCGAGGAATCA |
| | 3 | GGAAACAGTCTCTCAAACA |

426 **Supplementary Table 17. RT-qPCR primer sequence of select gene.**

| Gene name | Sequence (5'-3') |
|---|---|
| *OM* | F: TCACGGTTCGATCGCTTTGG |
| | R: TCCACTGCATCCAGTAGCCT |
| *LRR* | F: CCGAGCTTCACCTACAACGA |
| | R: CGGTGCAAGTTTCATGCCATC |

**Supplementary Table 18. The Sc-RNA sequencing information of *T. rubra* and *A. coerulea* medusa.**

| Species | Platform | Data | Number of captured cells | Number of selected cells |
|---|---|---|---|---|
| *T. rubra* | 10X Genomics | 120G | 28,607 | 22,245 |
| *A. coerulea* | BD Rhapsody | 120G | 37,671 | 18,936 |

**Supplementary Table 19. The Sc-RNA sequencing information of five stages of *T. rubra*.**

| Stage | Platform | Data | Number of captured cells | Number of selected cells |
|---|---|---|---|---|
| Planula (Pl) | 10X Genomics | 80G | 4,416 | 4,079 |
| Polyp (Po) | BD Rhapsody | 80G | 8,647 | 4,739 |
| Medusa (Me) | 10X Genomics | 120G | 28,607 | 22,245 |
| Four-leaf structure (Ff) | BD Rhapsody | 80G | 12,184 | 8,368 |
| Cyst (Cy) | BD Rhapsody | 80G | 7,416 | 5,523 |

**Supplementary Table 20. Sequences of the primers, related to ISH experimental procedures.**

| Species | Gene name | Gene ID | Sequence (5'-3') |
|---|---|---|---|
| *T. rubra* | *LOXHD1* | evm.model.Hic_asm_10.1109 | F: GGCATTGGTCCTGCATGGTA |
| | | | R: GCTGCTTGGTTGATAGTGGC |
| | *USH2A* | evm.model.Hic_asm_9.1159 | F: GTCAACGTGTCCTGGTCGAT |
| | | | R: TCCAGCCCAACATGAAACGA |
| *A. coerulea* | *LOXHD1* | Aco27060 | F: TTAGCACAGGTGGACTGGT |
| | | | R: ACCATCACACGACAAGGTGA |
| | *USH2A* | Aco20175 | F: ACAGTCGTTGGCTGCTCTAC |
| | | | R: AGGTGAGCTTTCTGGTGTCG |
| | *OMs* | Aco05836 | F: CGATGCCTGACCTAAGAGGC |
| | | | R: AAGACGCCTTGTGGCAGATA |
| | *CFAP141* | Aco15911 | F: TAAGGAACTTGAAGAGCAGTCTGT |
| | | | R: CAGTTGAGCTTGCCGAACC |
