# Supplementary material for: Genomic and single-cell analyses reveal genetic signatures of swimming pattern and diapause strategy in jellyfish
Source: Nat Commun. 2024 Jul 15;15:5936. doi: 10.1038/s41467-024-49848-z (PMC11250803; doi:10.1038/s41467-024-49848-z)
Supplement: Supplementary file 3 — Description of Additional Supplementary Files [file 41467_2024_49848_MOESM3_ESM.pdf]

Description of Additional Supplementary Files

Supplementary Data Legends:

Supplementary Data 1. 548 positive selected genes (PSGs) identified in T. rubra. The “Symbol” column represents the gene IDs of T. rubra(P-value<0.05).

Supplementary Data 2. dN/dS valve of the positive selected genes(PSGs) identified in T. rubra.

Supplementary Data 3. GO enrichment of positive selected genes(PSGs) in T. rubra (P value<0.05).

Supplementary Data 4. The RNA sequencing information of twotissues of four species.

Supplementary Data 5. The differentially expressed genes (DEGs) inthe rhopalia of three Scyphozoans and the tentacle bulb of T. rubra.

Supplementary Data 6. Enrichment of gene ontology of downregulated DEGs in the tentacle bulb of T. rubra (P-value <0.05).

Supplementary Data 7. The differentially expressed genes (DEGs) of the sensory organs and control tissues in each species.

Supplementary Data 8. Enrichment of gene ontology of upregulated/downregulated DEGs of the sensory organs in each species (P-value <0.05).

Supplementary Data 9. KEGG enrichment of DEGs of the sensory organs and control tissues in four jellyfish species (P-value<0.05).

Supplementary Data 10. The list of expanded/contracted gene families in jellyfish species. (P-value<0.05).

Supplementary Data 11. GO enrichment of expanded/contracted gene families in T. rubra (P-value<0.05).

Supplementary Data 12. The list of of gene losses in T. rubra (Pvalue<0.05). The statistical tests were two-sided and the adjustments were made for multiple comparisons.

Supplementary Data 13. GO enrichment of lost gene families in T. rubra (P-value<0.05).

Supplementary Data 14. The relative expression of target genes in the si-OM and si-LRR groups.

Supplementary Data 15. Cell atlas marker of medusa. The “gene” column represents the gene IDs of A. coerulea.

Supplementary Data 16. The different expressed genes (DEGs) of hair cell between T. rubra and A. coerulea. The “gene” column represents the gene IDs of A. coerulea.

Supplementary Data 17. GO enrichment of hair cells in T. rubra and A. coerulea (P-value<0.05).

Supplementary Data 18. The different expressed genes (DEGs) of neural cell between T. rubra and A. coerulea. The “gene” column represents the gene IDs of A. coerulea.

Supplementary Data 19. GO enrichment of neural cell in T. rubra and A. coerulea (P-value<0.05).

Supplementary Data 20. The different expressed genes (DEGs) of triated muscle between T. rubra and A. coerulea. The “gene” column represents the gene IDs of A. coerulea.

Supplementary Data 21. GO enrichment of striated muscle in T. rubra and A. coerulea (P-value<0.05).

Supplementary Data 22. Cell atlas markers for different developmental stages of the T. rubra.

Supplementary Data 23. KEGG enrichment analysis of cyst and planula in T. rubra.

Supplementary Data 24. GO enrichment analysis of cyst and planula in T. rubra (P-value<0.05).

Supplementary Data 25. The different expressed genes (DEGs) of T. rubra across different life stages.

Supplementary Data 26. Homologous gene comparison between T. rubra and A. coerulea.

Supplementary Software legends:

Supplementary Software 1. Scripts for extracting protein sequences of one-to-one orthologues from each orthogroup.

Supplementary Software 2. Scripts for individual protein alignments.

Supplementary Software 3. Scripts for gene loss analyses.

Supplementary Software 4. Scripts for integrate the datasets corresponding to the medusa.
