# Supplementary material for: Genomic and single-cell analyses reveal genetic signatures of swimming pattern and diapause strategy in jellyfish
Source: Nat Commun. 2024 Jul 15;15:5936. doi: 10.1038/s41467-024-49848-z (PMC11250803; doi:10.1038/s41467-024-49848-z)
Supplement: Supplementary file 6 — Reporting Summary [file 41467_2024_49848_MOESM6_ESM.pdf]

nature portfolio

Corresponding author(s): Qiang Lin, Zhijun Dong, Jianmin Zhao

Last updated by author(s): May 11, 2024

Reporting Summary

Nature Portfolio wishes to improve the reproducibility of the work that we publish. This form provides structure for consistency and transparency in reporting. For further information on Nature Portfolio policies, see our Editorial Policies and the Editorial Policy Checklist.

Statistics

For all statistical analyses, confirm that the following items are present in the figure legend, table legend, main text, or Methods section.

n/a	Confirmed	
☐	☒	The exact sample size (*n*) for each experimental group/condition, given as a discrete number and unit of measurement
☐	☒	A statement on whether measurements were taken from distinct samples or whether the same sample was measured repeatedly
☐	☒	The statistical test(s) used AND whether they are one- or two-sided<br>*Only common tests should be described solely by name; describe more complex techniques in the Methods section.*
☒	☐	A description of all covariates tested
☐	☒	A description of any assumptions or corrections, such as tests of normality and adjustment for multiple comparisons
☐	☒	A full description of the statistical parameters including central tendency (e.g. means) or other basic estimates (e.g. regression coefficient) AND variation (e.g. standard deviation) or associated estimates of uncertainty (e.g. confidence intervals)
☐	☒	For null hypothesis testing, the test statistic (e.g. *F*, *t*, *r*) with confidence intervals, effect sizes, degrees of freedom and *P* value noted<br>*Give P values as exact values whenever suitable.*
☒	☐	For Bayesian analysis, information on the choice of priors and Markov chain Monte Carlo settings
☒	☐	For hierarchical and complex designs, identification of the appropriate level for tests and full reporting of outcomes
☐	☒	Estimates of effect sizes (e.g. Cohen's *d*, Pearson's *r*), indicating how they were calculated

Our web collection on statistics for biologists contains articles on many of the points above.

Software and code

Policy information about availability of computer code

Data collection	All data collection used in this study are described in method section and supplementary information.
Data analysis	All open or commercial software and parameters used in this study are described in the method section of the manuscript. For Source data are provided with this paper. The codes used in the study were provided in the Supplementary information code (Supplementary Software 1-4), are available in our lab GitHub repository (https://github.com/Changhao051/Turritopsis-rubra). The data collection/data analysis software used in this study include: Diamond (v2.0.7.145), OrthoFinder (v2.2.7), MAFFT (v 7.475), RAxML (v 8.2.12), CAFÉ programme (v5.1.0), Eggnog 5.0, RepeatModeler (v2.0.2a) , EDTA (v1.9.7), DeepTE, RepeatMasker (v 4.1.2), Prank (v170427), PAML (v 4.9j), TBLASTN (v2.2.30), MEGA-X (v10.1.8), HISAT2 (v2.0.4), IBM SPSS Statistics 25, R (v.3.6.2), R package Seurat (v.4.0.6), R package Monocle 2 and R package Monocle 3.

For manuscripts utilizing custom algorithms or software that are central to the research but not yet described in published literature, software must be made available to editors and reviewers. We strongly encourage code deposition in a community repository (e.g. GitHub). See the Nature Portfolio guidelines for submitting code & software for further information.

Data

Policy information about availability of data

All manuscripts must include a data availability statement. This statement should provide the following information, where applicable:

- Accession codes, unique identifiers, or web links for publicly available datasets
- A description of any restrictions on data availability
- For clinical datasets or third party data, please ensure that the statement adheres to our policy

The whole-genome assemblies of T. rubra and A. coerulea have been deposited in the NCBI database under accession code PRJNA1005405. The raw reads of the RNA-seq of the four jellyfish have been deposited in the NCBI database under accession code PRJNA1010405. The raw reads of the single-cell RNA-seq of T. rubra and A. coerulea have been deposited in the NCBI database under accession code PRJNA1045549. Source data are provided with this paper.

Research involving human participants, their data, or biological material

Policy information about studies with human participants or human data. See also policy information about sex, gender (identity/presentation), and sexual orientation and race, ethnicity and racism.

Reporting on sex and gender	Sex was not considered in this study as it is irrelevant to the topic of the study.
Reporting on race, ethnicity, or other socially relevant groupings	n/a
Population characteristics	n/a
Recruitment	n/a
Ethics oversight	n/a

Note that full information on the approval of the study protocol must also be provided in the manuscript.

Field-specific reporting

Please select the one below that is the best fit for your research. If you are not sure, read the appropriate sections before making your selection.

☒ Life sciences ☐ Behavioural & social sciences ☐ Ecological, evolutionary & environmental sciences

For a reference copy of the document with all sections, see nature.com/documents/nr-reporting-summary-flat.pdf

Life sciences study design

All studies must disclose on these points even when the disclosure is negative.

Sample size	Two animals - one medusa of T.rubra and one medusa of A. coerulea - were used for whole-genome sequencing. 16 animals - four medusae of T. rubra, four medusae of A. coerulea, four medusae of C. quinquecirrha, four medusae of R. esculentum - were used for transcriptome analysis. For scRNA-seq library preparation, ~200 planulae, ~ 30 polyps, 5 four-leaf structures, 10 cysts, and one medusa of T. rubra and one medusa of A. coerulea were uesed. 160 polyps of A. coerulea were used for RNA interference experiment. Four animals - two medusa of T.rubra and two medusa of A. coerulea - were used for scanning electron microscopy. 16 medusa of T.rubra and 32 ephyrae of A. coerulea - were used for in situ hybridisation.
Data exclusions	No data was excluded in this study.
Replication	For each sample, scanning electron microscopy was conducted twice. In the RNAi experiment, four polyps of each group were collected for morphological observation. The micrographs of the in situ experiment were captured twice. In the in situ hybridisation experiment, eight replicates were set up for each gene in both species. Micrographs of each developmental stage of T. rubra were taken three times. No statistical method was used to predetermine sample size. No data were excluded from the analyses.
Randomization	At the end of the RNA interference experiment, four polyps were randomly selected for morphological observation, and the remaining 36 polyps of A. coerulea in each group were randomly divided into three biological replicates to extracted RNA for RT-qPCR.
Blinding	Blinded analyses were not performed in this study. RNA-interfering jellyfish identified using RT-qPCR were collected and observed morphologically using microscopy.

Reporting for specific materials, systems and methods

We require information from authors about some types of materials, experimental systems and methods used in many studies. Here, indicate whether each material, system or method listed is relevant to your study. If you are not sure if a list item applies to your research, read the appropriate section before selecting a response.

Materials & experimental systems

n/a	Involved in the study
☒	☐ Antibodies
☒	☐ Eukaryotic cell lines
☒	☐ Palaeontology and archaeology
☐	☒ Animals and other organisms
☒	☐ Clinical data
☒	☐ Dual use research of concern
☒	☐ Plants

Methods

n/a	Involved in the study
☒	☐ ChIP-seq
☒	☐ Flow cytometry
☒	☐ MRI-based neuroimaging

Animals and other research organisms

Policy information about studies involving animals; ARRIVE guidelines recommended for reporting animal research, and Sex and Gender in Research

Laboratory animals	160 polyps, 32 ephyrae and 6 medusae of A. coerulea, four medusae of C. quinquecirrha, four medusae of R. esculentum that cultured in the laboratory.
Wild animals	This study did not include wild animals.
Reporting on sex	Sex was not considered and this information was not collected in this study as it is irrelevant to the topic of the study.
Field-collected samples	~ 200 adult medusae of T.rubra were collected from waters near Yantai and Dongying between May and August 2021 and transported back to laboratory aquariums for cultivation.
Ethics oversight	All animal experiments were conducted per the guidelines and approval of the Yantai Institute of Coastal Zone Research, Chinese Academy of Sciences (2023-KJ-LL-002).

Note that full information on the approval of the study protocol must also be provided in the manuscript.

Plants

Seed stocks	n/a
Novel plant genotypes	n/a
Authentication	n/a
